# Antibacterial Activity of Nanoemulsions Prepared with Essential and Seed Oils Against Isolated Bacteria from Rainbow Trout (*Oncorhynchus mykiss*)

**DOI:** 10.3390/foods15132340

**Published:** 2026-07-02

**Authors:** Francisco Emilio Argote-Vega, Johannes Delgado-Ospina, Zully Jimena Suárez-Montenegro, Esteban Hernán Arteaga-Cabrera, Clemencia Chaves-López, José Ángel Pérez-Álvarez

**Affiliations:** 1 IPOA Research Group, Centro de Investigación e Innovación Agroalimentaria y Agroambiental de la UMH (CIAGRO), Miguel Hernández University, 03202 Orihuela, Spain; 2 Grupo de Investigación Biotecnología, Facultad de Ingeniería, Universidad de San Buenaventura Cali, Carrera 122 # 6-65, Cali 76001, Colombia; jdelgado1@usbcali.edu.co; 3 Grupo Tecnologías Emergentes en Agroindustria, Facultad de Ingeniería Agroindustrial, Universidad de Nariño, Calle 18 # 46-1 a 46-99, Pasto 522001, Colombia; zully.suarez.montenegro@udenar.edu.co; 4 Departamento de Alimentos, División de Ciencias de la Vida, Campus Irapuato-Salamanca, Universidad de Guanajuato, Carretera Irapuato-Silao km 9, Irapuato 36500, Guanajuato, Mexico; eh.arteagacabrera@ugto.mx; 5 Department of Bioscience and Technology for Food, Agriculture and Environment, University of Teramo, Via R. Balzarini 1, 64100 Teramo, Italy; cchaveslopez@unite.it

**Keywords:** essential oils, seed oils, nanoemulsions, antibacterial activity, fish, avocado, pumpkin, basil, mandarin, eucalyptus

## Abstract

This study investigated the inhibitory effect of nanoemulsions (NEs) derived from various essential oils (EOs) and seed oils (SOs) against pathogenic bacteria isolated from rainbow trout (*Oncorhynchus mykiss*). The EOs of eucalyptus, mandarin, and basil were extracted by hydrodistillation, while the SOs of avocado and pumpkin were extracted by supercritical fluids. GC–MS analysis determined the chemical composition, revealing that limonene (70.88%), eucalyptol (57.85%), and camphor (24.61%) were the main components of the EOs. The SOs were rich in palmitic acid (avocado) and linoleic acids (pumpkin) and contained phytosterols such as β-sitosterol and stigmasterol. Avocado seed oil had the highest total phenolic content and antioxidant activity. Eight stable NEs, prepared from individual and mixed oils, were tested against *Salmonella enterica* subsp. salamae, *Escherichia coli*, *Klebsiella variicola*, *Bacillus oceanisediminis*, and *Bacillus thuringiensis*. Most NEs were effective against *E. coli* and *B. oceanisediminis*, with an additive effect for SOs mixtures and a minimum inhibitory concentration of 0.53 μL/mL (*E. coli*), 0.53 μL/mL (*B. oceanisediminis*), and 2.13 μL/mL (*B. thuringiensis*). The bacteriostatic and bactericidal activity was 1.62 and 6 h with *E. coli* and NE Mix AP. These findings suggest that nanoemulsions containing SOs are promising candidates for controlling bacterial contamination in fishery products.

## 1. Introduction

Foodborne outbreaks (FBOs) represent a critical global health challenge, causing an estimated 600 million illnesses and 420,000 deaths annually [1]. Microbiological pathogens, particularly bacteria such as *Salmonella* spp., *L. monocytogenes*, *K. pneumoniae*, *B. cereus* and *E. coli*, are the primary etiological agents linked to severe health complications and significant economic losses [2]. In 2023, the European Food Safety Authority (EFSA) reported a 20.3% increase in FBOs associated with “fish and fishery products,” with *S. enteritidis* identified as a predominant pathogen [3,4].

Fish products, such as rainbow trout (*Oncorhynchus mykiss*), are highly susceptible to microbial spoilage due to their high protein and fat content, which facilitates the growth of both pathogenic and spoilage bacteria (e.g., *Pseudomonas*, *Shewanells*, *Psychrobacter*, *Lactococcus*, and *Acinetobacter*) [5,6,7,8]. Furthermore, the presence of *E. coli* in these products serves as a critical indicator of fecal contamination and potential public health risks [9]. This vulnerability is exacerbated by the global emergency of antimicrobial resistance (AMR) driven by the indiscriminate use of antibiotics in food industries, necessitating the development of alternative natural antimicrobial strategies [10,11,12].

Rainbow trout (*Oncorhynchus mykiss*) has become a strategic hydrobiological resource in Colombia, with its production and marketing heavily concentrated in the country’s inland and Andean regions. As the country’s per capita fish consumption has undergone historical growth—reaching 11.4 kg by 2025—the volume of fish mobilized through these regional distribution chains is larger than ever [13]. However, this commercial dynamism contrasts with hygienic deficiencies at local retail points, where the lack of good manufacturing practices and frequent cold chain breakages promote pathogen proliferation. In this scenario of high demand within the country’s interior, ensuring food safety becomes critical. This context fully justifies the evaluation of effective and sustainable preservation alternatives.

Essential oils (EOs) and seed oils (SOs) have emerged as potent natural alternatives to synthetic preservatives due to their high concentration of bioactive compounds, such as phenols, flavonoids, terpenoids, acids, saponins, tannins, and steroids, which exhibit significant antimicrobial and antioxidant activities [14,15,16,17]. However, the lipophilic nature of these compounds often limits their stability and bioavailability. Nanoemulsions offer a technological solution to these limitations, inhibiting lipid peroxidation in food and enhancing the contact between bioactive substances and bacterial membranes [18,19]. Nanoemulsions offer a technological solution to these limitations; they contact between bioactive substances and bacterial membranes While the efficacy of EOs from eucalyptus, citrus and basil, and in extending the shelf life of fish has been documented [19,20,21], research regarding the in situ and in vitro application of avocado and pumpkin seed oils remains comparatively scarce, with some research reporting that seed extracts exhibit antibacterial activity [22,23,24].

This study evaluates the in vitro antibacterial efficacy of nanoemulsions formulated with EOs (*Eucalyptus globulus*, *Citrus reticulata*, and *Ocimum basilicum*) and SOs (*Persea americana* Mill. and *Cucurbita maxima*), both individually and in specific mixtures (Mix EBM: A mix of essential oils of eucalyptus leaves, basil leaves, and mandarin peels; A mix AP: A mix of seed oils of avocado and pumpkin seeds; Mix EBM + AP: A mix of essential and seed oils). The antimicrobial potential was assessed against a total panel of 15 strains of pathogenic and opportunistic bacterial species, including *S. enterica*, *E. coli*, *K. variicola*, *B. ocanisediminis* and *B*. *thuringiensis*, all specifically isolated from rainbow trout to ensure ecological and industrial relevance. The practical applications of this research are intended for the aquaculture and post-harvest sectors, particularly in traditional open markets and regional distribution networks within the Andean interior, where maintaining a strict cold chain remains challenging. By evaluating nanoemulsions and pure oils against locally relevant foodborne isolates, this study bridges the gap between natural antimicrobial research and sector food safety needs, offering a scalable, bio-based preservation strategy to protect public health amid rising fish consumption in Colombia.

## 2. Materials and Methods

### 2.1. Extraction of Oils

Eucalyptus leaves, basil leaves, and mandarin peels were dried at 50 °C, while pumpkin and avocado seeds were dried at 60 °C. Moisture content was determined according to the AOAC standard [25]. Essential oils (EOs) were obtained by hydrodistillation (water/material: 5:1; temperature: 90 ± 0.30 °C; time: 3–4 h [26]). The seed oils (SOs), with an approximate particle diameter of 0.6 mm, were extracted employing supercritical CO_2_ with a Waters™ SFE-500 Thar system (Waters Corporation, Milford, MA, USA), at a pressure of 25 kPa (pumpkin) and 150 MPa (avocado) under the following conditions: time: 120 min; CO_2_ flow rate: 0.25 L/min; and cosolvent: hexane. The samples of EOs and SOs, obtained in triplicate, were dried with anhydrous sodium sulfate and stored at 4 ± 0.5 °C in dark vials. The oil yield was determined per Equation (1).

Yield (%) = (oil weight in g)/(weight of plant material in g) ∗ 100(1)

### 2.2. Determination of Oils Quality Parameters

The quality parameters of the EOs and SOs were evaluated following the methodology described by AOAC [25]. The parameter density, refractive index, iodine value, acidity, saponification, and peroxide indices were assessed in triplicate.

### 2.3. Chemical Composition of Essential Oils

The composition of EOs was analyzed by a gas chromatograph–mass spectrometer (GC–MS; Shimadzu GCMS-QP2010S, Kyoto, Japan), DB5-MS column, 30 m × 0.25 mm × 0.25 µm (Chrom Tech Inc., Apple Valley, CA, USA) and a selective mass detector at full scan mode (Agilent Technologies, MSD5970, Hewlett-Packard, Geneva, Switzerland) employing SPME according to Chaves-López et al. [27]. The CAR/PDMS fiber (75 mm), was conditioned at 270 °C and exposed to headspace for 40 min at 50 °C prior to injection. Injector and FID temperatures were 250 °C, detector 220 °C, with helium as the carrier gas (1 mL/min). The oven program was 140 °C (2 min), ramp to 200 °C at 10 °C/min, then to 250 °C at 15 °C/min, held 5 min. Compounds were identified by NIST library comparison, and relative quantities calculated from GC peak area without correction factors.

### 2.4. Fatty Acid Profile of Seed Oils

The fatty acids were esterified with the Christie method [28] and the fatty acid methyl esters (FAMEs) were analyzed by GC–MS as described by Ordoñez-Lozada et al. [29]. The seed oils were saponified, then esterified, extracted with a NaCl and hexane solution, and subsequently centrifuged. A 1 µL sample was injected into the gas chromatograph coupled with a mass spectrometer (QP2010, Shimadzu, Kyoto, Japan). Identification was based on retention time and spectra compared with Supelco 37 FAME CRM47885 (Merck Life Science, Milano, Italia). Results were expressed as relative peak area percentages based on triplicate samples.

### 2.5. Analysis of Sterols in Seed Oils

The sterols present in SOs were determined with the AOCS method [30]. At the start, 1 octacosanol and 5 cholestane (internal standards) were added. Hydrolysis was performed by alkaline saponification [29], and the unsaponifiable fraction was isolated by silica gel plate chromatography (hexane/diethyl ether, 65:35 *v*/*v*). The sterol bands were visualized under UV light, and silylation was performed according to Lombardi et al. [31]. GC analysis was performed on a DANI GC1000 DPC (Alltech, Milan, Italy). Helium was used as the carrier gas (1.2 mL/min). The percentages of sterols were calculated from the peak areas with a correction factor of 1, as described by Laakso [32]. The results were expressed in mg/100 g based on triplicate samples.

### 2.6. Tocopherols Analysis by HPLC-FL

The tocopherols present in SOs were determined by HPLC-FL [29]. The samples were diluted, filtered, and injected into an HPLC system (Shimadzu, Kyoto, Japan) equipped with a fluorescence detector (RF 20A). Separation was performed at 25 °C on a C18 column (4.6 mm × 150 mm, 5 µm) (Thermo Scientific, Waltham, MA, USA). Mobile phase: acetonitrile: methanol: isopropanol, 50:40:10 (0–10 min), 30:65:5 (10–33 min), held for 12 min, and then returned to the initial conditions (5 min). Flow rate: 1 mL/min. Excitation/emission wavelengths: 290/330 nm. Identification was based on retention time and the spectra of the standard samples; quantification was performed with calibration curves for α, δ, and γ-tocopherol. Results are expressed in mg/100 g of oil based on triplicate samples.

### 2.7. Preparation of Nanoemulsions

Eight oil-in-water (O/W) nanoemulsions (NEs) were formulated: three containing essential oils (eucalyptus (EEO), mandarin (MEO), and basil (BEO)), two containing seed oils (avocado (ASO) and pumpkin (PSO)), and three mixtures (essential oils (Mix EBM), seed oils (Mix AP), and a combined mixture (Mix EBM + AP)). The MEO and EEO NEs were prepared with the method proposed by [19] with a sonication device (Branson SFX 550, 2 min, 18% amplitude, 188 W). The BEO NE was prepared according to the method described in [21]. The ASO and PSO NEs were prepared according to the method in [29]. The mixtures were prepared as follows: EBM mixture (20% EEO, 50% BEO, 30% MEO), according to [19,21]; AP mixture (60% ASO, 40% PSO), as described in [29]; and EBM + AP mixture (10% EEO, 25% BEO, 15% MEO, 30% ASO, 20% PSO) combining the three cited methods [19,21,29]. The concentrations were selected based on the solubility limits of the nanoemulsions and on concentration ranges (*v*/*v*) reported in previous studies for similar formulations as reported above. Thermodynamic stability was evaluated following Sugumar et al. [33] by centrifugation (3000 rpm, 30 min), heating–cooling cycles (4–40 °C), and freeze–thaw cycles (−21–25 °C), assessed at 0–180 days of storage (4 °C, amber vials). Stable formulations were used to prepare stocks (20 µL/mL) and serial dilutions (10–0.53 µL/mL) and were stored at 4 °C for minimum inhibitory concentration (MIC) determination and physical stability characterization.

#### Characterization of Nanoemulsions

The droplet size, polydispersity index (PDI), and ζ potential of the eight stock NEs were measured at 0, 90 and 180 days of storage with a Zetasizer ZS90 (Malvern Panalytical, Worcestershire, UK) with DTS0012 and DTS1070 cuvettes. To minimize multiple scattering, samples were diluted in double-distilled water at ratios of 1:100 (*v*/*v*) for essential oil-based NEs [19] and 1:10 for seed oil-based NEs [29]. Measurements were conducted at 25 °C with a 90° scattering angle, 120 s equilibrium time, and ultrapure water as solvent. Each determination was performed in triplicate. The pH of the formulations was also recorded with a calibrated pH meter NTPHM (Nova Técnica, Sao Paulo, Brazil).

### 2.8. Antioxidant Capacity and Total Phenolic Content of Pure Oils and Nanoemulsions

The antioxidant activity of EOs and SOs, as well as NEs, was determined with the DPPH and ABTS^•+^ assays, while total phenolic content (TPC) was determined by the Folin–Ciocalteu method.

#### 2.8.1. DPPH Assay

The radical scavenging activity was determined following Delgado-Ospina et al. [34] with modifications. Results were expressed as µmol Trolox equivalents per mL of oil or NE, based on calibration curves. All analyses were performed in triplicate.

#### 2.8.2. ABTS^•+^ Assay

The ability of the antioxidant capacity was also assessed with the ABTS^•+^ decolorization method [34]. Antioxidant activity was calculated relative to Trolox standards and expressed as µmol Trolox equivalents per mL of oil or NE. Measurements were conducted in triplicate.

#### 2.8.3. Total Phenolic Content

The total phenolic content (TPC) of pure oils and NEs was determined with the Folin–Ciocalteu method as described by Delgado-Ospina et al. [35]. Quantification was performed against a gallic acid calibration curve, and results were expressed as mg gallic acid equivalents (GAEs)/mL of oil. The same procedure was applied to nanoemulsions, with all analyses conducted in triplicate.

### 2.9. Inhibitory Activity of NEs and Oils

Strains of *Escherichia coli* (TJC3, TJC7, TJC10), *Bacillus oceanisediminis* (TJC1, TJC18, TJC8), *Bacillus thuringiensis* (TJC2, TJC20, TJC25), *Salmonella enterica* subsp. *salamae* (TJC5, TJC13, TJC21), and *Klebsiella variicola* (TJC15, TJC19, TJC11) were isolated under aseptic conditions from the skin surface (peel) of rainbow trout (*Oncorhynchus mykiss*) sourced from local markets in Pasto, Colombia. The isolation process strictly followed standard ISO procedures, specifically ISO 4332 [36] on PCA for total viable count, ISO 21528-2 [37] on VRBG for Enterobacteriaceae, ISO 7251 [38] for *E. coli*, and ISO 6579 [39] for the detection of *Salmonella* spp., with final strain identification performed as described by [40]. All isolates were subsequently preserved in the University of Teramo’s culture collection at −70 °C in nutrient broth supplemented with 20% glycerol. These specific strains were then retrieved and utilized to evaluate and compare the antimicrobial activity of both pure oils (EOs and SOs) and their respective nanoemulsions (NEs).

Prior to assays, cultures were grown in tryptic soy broth (TSB) with 0.1% glucose at 37 °C for 24 h, harvested by centrifugation (6000 rpm, 5 min), washed with PBS (50 mM, pH 7), and standardized to 0.1 OD (~5 × 10^5^ cells/mL). Antimicrobial activity was determined by the microdilution method [41,42] in 96-well plates (350 µL volume). Each well received 200 µL of bacterial suspension and 100 µL of NE or oil. Pure oils were dissolved in PBS–Tween 80 (1:2, *v*/*v*) to obtain a stock solution (60 µL/mL), from which serial dilutions (10–50 µL/mL) were prepared. NEs were tested at concentrations of 10–0.53 µL/mL. Plates were incubated at 37 °C for 48 h, and minimum inhibitory concentration (MIC) was determined using 2,3,5-triphenyltetrazolium chloride (TTC, 1% *w*/*v*), with color change indicating metabolic activity. Minimum bactericidal concentration (MBC) was established by plating 100 µL from wells without visible growth onto TSA and incubating at 37 °C for 48 h. The MBC was defined as the lowest concentration at which no colonies developed. All assays were performed in triplicate, and inhibitory activity was assessed at day 0 of storage.

### 2.10. Calculation of the Fractional Inhibition Concentration Index (FICI)

The FICI was calculated for the NEs corresponding to the Mix EBM and Mix AP mixtures, as well as to their combination, Mix EBM + AP, following the method of Davidson and Parish [43]. The fractional inhibitory concentrations were obtained with Equation (2):

FIC_A_ = MIC_A+B_/MIC_A_, FIC_B_ = MIC_B+A_/MIC_B_, FICI = FIC_A_ + FIC_B_. (2)

The interpretation of the FICI was based on the criteria proposed by Odds [44], classifying the interactions as antagonistic (FIC > 4), additive (0.5 < FIC < 4), or synergistic (FIC ≤ 0.5).

### 2.11. Inactivation Kinetics

Based on the microdilution results, inactivation kinetics were performed with Mix AP-NE. The assay followed López et al. [45] with modifications. Bacterial inocula were standardized to 1 × 10^6^ CFU/mL (McFarland scale), and aliquots of NE were added to 9 mL of suspension to reach the MIC. Cultures were incubated at 37 °C for 48 h, and samples were taken at 0, 1, 2, 4, and 6 h for colony counting by serial dilution on nutrient agaracteriostatic and bactericidal effects were defined according to Scorneaux et al. [46] as reductions of <3 log CFU/mL and ≥3 log CFU/mL, respectively, relative to the initial inoculum. All experiments were conducted in triplicate.

### 2.12. Kinetic Modeling

The inactivation kinetics of the Mix AP-NE against *B. oceanisediminis*, *E. coli*, and *B. thuringiensis* were fitted to a Weibull distribution model following Salvia-Trujillo et al. [47]. The survival fraction of bacteria was expressed as follows:

(3) $$S(t)=exp\;{\left(-(t/\beta \right)}^{\alpha })$$

Applying the natural logarithm with Equation (4)

(4) $$lnS(t)=ln\;\left(\frac{N}{N_{0}}\right)={-(t/\beta )}^{\alpha }$$

where S(t) represents the survival fraction (N/N_0_), t is the reaction time (h), β is the scale parameter indicating the time required for the first logarithmic reduction, and α is the shape parameter describing the curve trend. Experimental data were linearized according to the model Equation (5), and regression coefficients were calculated. Model accuracy was assessed with the precision factor (*Af*), as described in Equation (6).

(5) $$ln\;\left[-ln\;\left(\frac{N}{N_{0}}\right)\right]=\alpha \ln t-\alpha \ln\beta$$

(6) $$A_{f}=10^{\frac{\sum \left|log(\frac{predicted}{observed})\right|}{n}}$$

### 2.13. Statistical Analysis

Data were analyzed employing Statgraphics^®^ Centurion XVII (Statgraphics Technologies Inc., Virginia, The Plains, VA, USA). Differences in quality parameters between essential and seed oils, as well as antioxidant capacity (oils vs. nanoemulsions), and the physical stability of nanoemulsions prepared with SOs and EOs, were assessed by one-way ANOVA followed by Tukey’s HSD test at a 5% significance level. For non-parametric data, comparisons were performed with the Kruskal–Wallis test. Results from moisture, yield, compositional analyses (essential oils, fatty acids, sterols, tocopherols), fractional inhibitory concentration index (FICI), and time-kill kinetics of Mix AP-NE are reported as mean ± standard deviation (SD).

## 3. Results

### 3.1. Extraction of Essential and Seed Oils

Moisture content was lower in SOs than in EOs, as time and temperature, drying method, type and shape of the leaf or material, size, porosity, thickness, initial water content, and coatings, among other factors, influence the final moisture content of plant material after drying and conditioning operations [48].

The yields of EOs and SOs are shown in Table 1, with higher yields for EEO and PSO.

Other authors with EEO reported yields of 1.35% [49] and between 0.59–1.24% [50]. With MEO, researchers [26,51] obtained yields of approximately 0.22% (2.5 mL/kg) and 3.19%. A study [21] with BEO achieved a 1% yield. On the other hand, ASO, according to [52], achieved a yield of 4.3% (Mexican) and 6.9% (Brazilian) with the Hass variety. Artica-Malqui et al. [53] reported a yield of 28.34% with PSO. These results suggest that SFE offers advantages in terms of higher yield, selectivity, and solubility of organic compounds [54]. SOs are composed mainly of fatty acids, sterols, tocopherols, polyphenols, saponins, and tannins [23,55,56], among others, unlike EOs, which contain heat-sensitive and highly volatile compounds [57]. Factors such as the plant, cultivar, growing conditions, harvest season, seed ripening stage, extraction method, and tissue type influence the composition and yield of essential oils [58].

### 3.2. Quality Parameters of Essential and Seed Oils

As shown in Table 2, the refractive indices did not show significant differences (*p* > 0.05) between the SOs and the EOs. This was somewhat unexpected, as refractive index is generally influenced by molecular structure, chain length, and degree of unsaturation of the fatty acids [59]. However, the values obtained for both oil types fell within the typical ranges reported for vegetables and EOs (1.46–1.47) and the lack of statistical difference may be explained by overlapping characteristics among the samples tested. In contrast, density was significantly affected by plant species, chemical composition, and the oil extraction method, among other factors [60]. The acid, peroxide, iodine, and saponification indices showed significant differences (*p* < 0.05) for SOs. Acidity values were below the limit of 4 mg KOH/g of oil [61]. This limit is a key quality criterion for edible oils, in fact values under 4 mg KOH/g indicate low free fatty acid content, meaning the oils have not undergone significant hydrolytic rancidity and are chemically stable. Similarly, peroxide values remained below 15 mEq O_2_/kg of oil, which is within the acceptable limit for fresh oils. Based on these two parameters, the seed oils showed no alteration, are stable, and are suitable for consumption. According to the iodine value, SOs are classified as non-drying oils (iodine value < 100), indicating the presence of unsaturated fatty acids but at levels insufficient for film-forming properties. In addition, the saponification index suggested that ASO has a higher average molecular weight than PSO, comparable to that of peanut and mustard oils which are used in industrial, pharmaceutical, and food applications [62]. Comparison with the literature reveals both agreement and variability. For example, for PSO, previous studies reported lower acidity (0.15 mg KOH/g of oil), iodine value (75.63 g I_2_/100 g of oil), and saponification value (100.68 mg KOH/g of oil) than of our findings [29]. Ali et al. [63] determined a higher peroxide value (12 mEq of active O_2_/kg of oil) for PSO; such differences may be attributed to variations in the crop chemical composition, extraction conditions, and storage. For ASO, Adaramola et al. [64] reported high acidity (4.51 mg KOH/g oil) but lower peroxide values (2.40 mg O_2_/kg oil), saponification (35.76 ± 0.07 mg KOH/g of oil), and iodine (23.5 g of I_2_ per 100 g of oil) compared with the results of this study. Table S1 shows the results of the statistical analysis of the quality indexes (Supplementary Materials).

### 3.3. Composition of Essential and Seed Oils

Table 3 shows the main isoprenoids present in essential oils. MEO contained predominantly limonene, γ-terpinene, and linalool, which are typical of citrus fruits such as oranges, lemons, and grapefruits. Compounds rich in α-pinene, linalool, and thymol are known for their significant antimicrobial activity. The limonene concentration ranges between 32–98%, depending on the variety [60]. EEO was characterized by chemotypes rich in eucalyptol and α-pinene. Previous studies have reported variable compositions, with higher percentages of eucalyptol (85.40%) and lower percentages of α-pinene (5.40%) [50,65]. BEO showed significant levels of camphor and eucalyptol. Although compounds such as linalool and eugenol are present in lower concentrations, they contribute synergistically to antimicrobial activity. Another study reported higher concentrations of eugenol (71.3%), α-cubebene (6.4%), and linalool (4.1%) [66]. Pérez-Cordero et al. [67] found lower levels of eucalyptol in the essential oil of *O. basilicum* from three municipalities in Colombia, ranging from 0.07% to 0.28%. Variations in the abundance of these compounds are influenced by climatic and geographical factors, the parts of the plant used, drying conditions, extraction methods, and the analytical techniques employed [68,69].

Table 4 shows the fatty acid composition of the SOs. PSO was rich in linoleic acid, followed by oleic, palmitic, and stearic acids. Researchers [29] have reported the presence of linoleic acid (28.45–42.4%), palmitic acid (13.6–15.9%), oleic acid (28.9–39.4%), and stearic acid (11.5–15.7%) in the varieties *C. maxima*, *C. moschata*, and *C. pepo*. The ASO analysis highlighted the abundance of palmitic, linoleic, oleic, and stearic acids. A study [52] on Brazilian and Mexican Hass varieties identified significant amounts of oleic acid (30.2–32.9%), linoleic acid (44.0–45.0%), and palmitic acid (16.4–19.6%). Differences in the profile and content of fatty acids in seed oils are likely influenced by genetic factors, geographic and cultivation origin, climate, and the natural environment [70].

Stigmasterol, β-sitosterol, and γ-tocopherol were identified in the PSO (see Table 5). Significant amounts were found in [29] for γ-tocopherol (359.56 mg/100 g), similarly, Artica-Malqui et al. [71] identified β-sitosterol (19.50 mg/100 g), while Dotto and Chacha [72] reported γ-tocopherol (97.15–893 mg/kg), stigmasterol (38.7–134.83 mg/kg), and β-sitosterol (12.08–100 mg/kg). In ASO, the compound found in the highest concentration was stigmasterol, compared with β-sitosterol, α-tocopherol, and campesterol. The researchers [73] also identified β-sitosterol (19.17 mg/kg) as one of the most abundant compounds. The concentration of bioactive compounds in SOs is influenced by environmental conditions such as climate, geographic origin, and genetic factors [74,75].

### 3.4. Thermodynamic Stability of Nanoemulsions

The results of the thermodynamic stability tests are presented in Table 6; the PSO, BEO, and EEO nanoemulsions (NEs) exhibited low stability after centrifugation. O/W nanoemulsions are more susceptible to phase separation due to the lower density of oil when compared with water, which results in reduced Brownian motion [76]. During the heating and cooling cycle, no changes were observed for up to 90 days at 4 °C; however, at 40 °C, phase separation was observed in PSO, BEO, MEO, Mix AP, and EEO after 180 days of storage; this behavior was the same in the freeze–thaw stress test. Some authors attribute this to the coalescence of droplets due to the dehydration of the main surfactant groups [77]. The higher stability of Mix EBM compared with individual nanoemulsions is attributed to synergistic pH buffering, enhanced surface charge, and complementary antioxidant activities among eucalyptus, basil, and mandarin oils. In contrast, Mix AP did not exhibit such synergy because the inherently unstable pumpkin oil dominated the mixture, lowering pH and reducing zeta potential, despite the high stability of avocado oil alone.

### 3.5. Physical Characterization of Nanoemulsions

**Figure 1:**
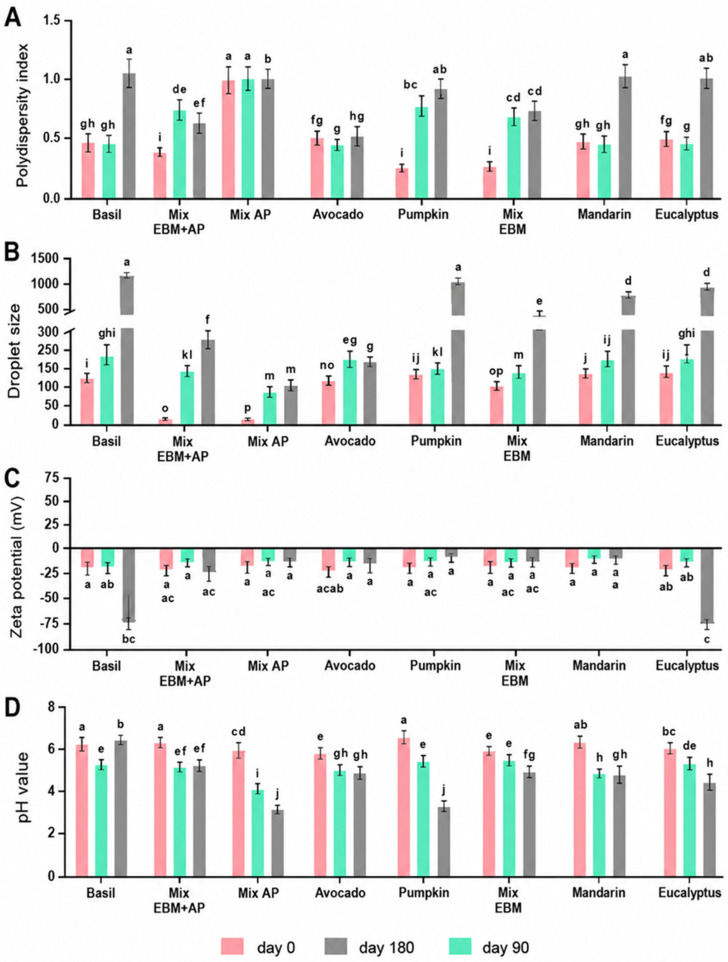
Characteristics of nanoemulsions during storage: Polydispersity index (**A**), changes in droplet size (**B**), particle charge (**C**), and pH (**D**) of nanoemulsions made with essential oils and seed oils during storage at 4 °C at day 0, day 90 and day 180. Data are presented as bars representing mean ± SD of three replicates. Different letters indicate significant differences according to Tukey’s post hoc test (HSD) at *p* < 0.05.

On day 0 of storage, as shown in Figure 1B, Mix AP, Mix EBM+AP, and ASO had the smallest mean droplet sizes (Ds), with significant differences (*p* < 0.05) compared with the other NEs, among which NE EEO had the highest mean Ds. At 90 days, NEs indicated Ds ranging from 59.43–204.80 nm for Mix AP and ASO, respectively, with significant differences (*p* < 0.05) among all. Researchers have reported that the most stable NEs are those with particle sizes below 200 nm [78,79]. However, at 180 days, Mix AP, ASO, Mix EBM+AP, and Mix EBM showed the lowest sizes with significant differences (*p* < 0.05). On the other hand, all NEs presented significant differences (*p* < 0.05) when comparing Ds between the time points of 0, 90, and 180 days; however, between 0 and 90 days, MEO and PSO exhibited greater stability without significant differences (*p* > 0.05). The droplet size and polydispersity index depend on the geometry of the vessel in which sonication takes place, as well as on the energy delivered by the ultrasonic probe [19,80]. Similarly, the efficiency of sonication and cavitation is influenced by the distance from the solution and the vessel diameter, and ultrasonic energy is related to the propagation distance [81,82,83]. The oil–water interfacial tension and the external exposure of the droplet size are also related to the type and concentration of the surfactant [84]. Studies with low-energy methods have shown that droplets smaller than 200 nm with low polydispersity can be obtained and remain stable for up to 30 days of storage [85].

Regarding the values of the polydispersity index (PDI) on day 0 of storage, significant differences were observed (*p* < 0.05), particularly between the PSO and EEO NEs, with values ranging from 0.24 to 0.52, respectively, while at 90 days, they showed values of 0.43 for MEO and 1.00 for Mix AP, with significant differences (*p* < 0.05). After 180 days, the NEs reached a polydisperse distribution with levels between 0.62 and 1.00, except in the case of ASO, which maintained a stable PDI of 0.48. Significant differences (*p* < 0.05) were observed when comparing the 0, 90, and 180-day storage periods, except for ASO, which indicated no significant differences (*p* > 0.05) and exhibited stable behavior. Between 0 and 90 days, ASO, MEO, EEO, and BEO presented no significant differences (*p* > 0.05). The PDI indicates the stability and uniformity of the particle size distribution. A value close to 0 indicates a monodisperse distribution; conversely, values greater than 0.5 indicate a polydisperse distribution with low stability [86,87]. In this regard, the NE with the lowest polydispersity was ASO, and high polydispersity with a PDI of 1 for BEO, EEO, MEO, PSO, and Mix AP at 180 days of storage, which showed alterations in the thermodynamic stability tests (see Table 6 and Figure 1A).

Furthermore, in Figure 1C, which shows the zeta potential (Zp), no significant differences (*p* > 0.05) were observed among the NEs at 0, 90, and 180 days, with high dispersion of the BEO and EEO at the end of the experiment. No significant differences (*p* > 0.05) were observed when comparing Zp values between 0, 90, and 180 days of storage for the Mix EBM, ASO, and Mix EBM + AP, and between 0 and 90 days, in addition to those mentioned above, for BEO and EEO. NEs with a Zp greater than or equal to +30 mV or less than or equal to −30 mV indicate high stability, due to repulsive forces between the nanoparticles [76,88]. The type of surfactant, the adsorption of hydroxyl ions from the aqueous phase, the presence of residual ionic impurities, the free fatty acid content in the emulsifier and in the oil, and the composition related to the acidity index in vegetable oils can influence variations in Zp, with low acidity values being the most favorable [29,89].

As indicated in Figure 1D, significant differences (*p* < 0.05) were observed in the NEs on days 0, 90, and 180, with a significant decrease in pH on the last day of storage to approximately 3 for the Mix AP and PSO. When comparing pH between days 0, 90, and 180, all NEs presented significant differences (*p* < 0.05). The decrease in pH in this study corresponds to that described by [29], the pH of the aqueous phase influences the Zp and physical stability; an increase in the medium’s pH favors higher negative Zp values with an increase in charge repulsion between colloidal particles, which is not favorable for acidic media below or around pH 3. The results of the statistical analysis of the physical stability indicators can be found in Table S1. The Mix EBM was more stable over time, with a pH of 5.69 on day 0 and a Zp of −17.70, which varied to a pH of 4.95 and a Zp of −8.39 after 180 days of storage. In contrast, Mix AP showed a significant decrease in pH, dropping from 5.65 on day 0 to 3.15 after 180 days, which influenced the variation in Zp from −25.20 to −2.39, with a value close to zero indicating the low stability of the NE. Negative Zp values represent the negative electrical charge on the surface of the droplets, and the further they are from zero, the greater the repulsion, preventing their separation from the aqueous phase or agglomeration [76]. The components of Mix EBM exhibited similar individual behaviors, as evidenced by the variations in pH and Zp between 0 and 180 days for the individual NEs with BEO (pH: 6.29 to 6.34; Zp: −17.20 to −17.50), MEO (pH: 6.28 to 4.75; Zp: −24.60 to −7.87) and EEO (pH: 6.11 to 4.59; Zp: −22 to −11.20), whose pH and Zp variations were low compared with those of the PSO component in the individual NE (pH: 6.34 to 3.17; Zp: −26.90 to −2.52) of Mix AP, which in turn favored greater particle repulsion for Mix EBM, resulting in the stability of the nanoemulsion over time. In the case of Mix AP, the ASO component was more stable in the individual NE, due to its high phenolic content and high antioxidant activity (Table 7), in which it was present at a higher concentration when compared with that in the Mix AP mixture, which was 60%. Meanwhile, PSO accounted for 40% with lower antioxidant activity, which may have influenced the oxidation or hydrolysis of fatty acids due to the significant decrease in pH [88], as reflected in a Zp (−2.52) that indicated low stability on the last day of storage, not only for the individual NE, but also for Mix AP.

Among the possible causes of the significant variation observed in the NEs between 0 and 180 days of storage, in addition to those described in this chapter, the following are worth mentioning: the method used to prepare the NEs (for this study, these were high-energy homogenization and ultrasound), temperature fluctuations in the refrigeration equipment, changes in surfactants over the storage period, and oxidative or hydrolytic processes (particularly in NEs prepared with PSO), all of which could have influenced the aggregation of droplets without coalescence (flocculation) and their migration to the upper surface with cream formation (creaming). These phenomena were observed in thermodynamic stability tests (Supplementary Materials Figure S2) and have been extensively studied by other authors [76]. Most of the NEs remained stable for up to 90 days of storage, as indicated by the thermodynamic stability tests and the physical characterization parameters of droplet size, polydispersity index, Z-potential, and pH. Figure S1 (Supplementary Materials) shows the characterization curves of the nanoemulsions over the course of storage. The reference studies [19,21,29] used to prepare the NEs reported stability indicators that were significantly lower than those observed in this study during the first days of storage.

### 3.6. Antioxidant Capacity and Total Phenolic Content of Nano-Emulsions, Essential Oils and Seed Oils

The evaluation of the protective effect of EOs and SOs against reactive oxygen species and free radicals, with the ABTS^•+^ and DPPH methods, revealed significant differences (*p* < 0.05), with representative values for ASO, BEO, and PSO. The total phenolic content (TPC) of the oils also showed significant differences (*p* < 0.05), particularly for ASO and EEO. The NEs exhibited greater antioxidant capacity than that observed in the pure oils, with significant differences (*p* < 0.05) in the ABTS^•+^ and DPPH assays for ASO, BEO, and PSO. The correlation of ABTS^•+^ and DPPH with Ds for the NEs was moderately negative, with a value of −0.75, while a perfect positive correlation, with a value of 1, was observed between methods (ABTS^•+^, DPPH) and TPC. The significant differences (*p* < 0.05) and the increase in antioxidant activity of the NEs compared with the pure oils can be attributed to their ability to encapsulate active compounds in small droplets, which improves their bioavailability and protection against environmental factors [19] (see Table 7). The results of the statistical analysis of antioxidant activity are shown in Table S1.

One study [29] found that oil concentration, regardless of variety, is the factor that determines antioxidant capacity; in the case of *C. maxima*, this activity was attributed to the carotenoid lutein, as well as to polyphenols, phenolic acids, flavonoids, sterols, and tocopherols [90], with the latter being responsible for protecting lipids by eliminating peroxyl radicals [91], just as carotenoids do in the inactivation of other stages of chain propagation involving electronically excited molecules in an “extinction” process [92]. Researchers reported significant TPC and antioxidant activity in PSO and ASO extracts, with different techniques and varieties as influencing variables [75,93,94]. In the case of essential oils, antioxidant activity has been linked to polyphenols [50,95,96] and, in particular, to BEO, with phenolic acids such as caffeic, chicoric, caffeoyl, ferulic, and rosmarinic acids [97].

### 3.7. Antibacterial Activity of Nanoemulsions Against Bacteria Isolated from Rainbow Trout (Oncorhynchus mykiss)

The antibacterial activity of NEs is attributed to the small size of their oil particles and their consequently high surface tension, which can merge with and severely damage bacterial cell membranes, leading to cell death. Additionally, NEs enhance oil stability by reducing oxidation and degradation compared with free compounds, thereby enhancing their antibacterial action [98,99]. Table 8 presents the inhibitory effect of NEs and pure oils against rainbow trout bacteria.

#### 3.7.1. Antibacterial Activity of Nanoemulsions with Single Oils

Among the EO formulations, EEO NE was the least effective, showing a high MIC (10 μL/mL) against *E. coli* strains, comparable to its pure oil (40–50 μL/mL). In contrast, BEO NE exhibited a low MIC (2.13 μL/mL) against *E. coli*, *B. thuringiensis*, and *S. enterica*, and a high MIC (10 μL/mL) against *B. oceanisediminis.* The NE with MEO showed a low MIC (4.25 μL/mL) for *E. coli* strains versus a high MIC (10 μL/mL) for *B. oceanisediminis.* The pure basil and mandarin oils exhibited the same antimicrobial pattern as their respective NEs (see Table 8).

In EO-based NEs, antibacterial activity depends on oil type, species, and composition. Although gram-negative bacteria are generally considered more resistant than gram-positive bacteria, both can exhibit high resistance [100]. Previous studies have reported the efficacy of BEO NE against various pathogens, including *Aeromonas hydrophila*, *Aeromonas veronii*, *Pseudomonas fluorescens*, *Streptococcus agalactiae*, and *Saprolegnia parasitica*, (MICs 3–6 µL/mL) [101], as well as against *E. coli*, *Proteus mirabilis*, and *S. aureus* [102]. On the other hand, MEO is generally composed of D-limonene and has shown activity against *S. aureus*, *L. monocytogenes*, and *S. enterica* [103]. Additionally, ref. [19] has reported the in vitro antibacterial activity of an EEO NE against *E. coli*. In all cases the antibacterial activity is attributed to the main components that react with the phospholipids of the cell membrane, increasing its permeability [104].

In a previous work with biofilms [40], using the same bacterial strains employed in this study, we observed antibiofilm activity of *Ocimum basilicum* essential oil from Italy and from Colombia against these fish isolates. A related study [105] demonstrated that *Eucalyptus globulus* essential oil—which was functionalized with iron oxide nanoparticles (Fe_3_O_4_) within polylactic acid composite coatings—significantly inhibited biofilm formation and maturation in major pathogens such as *Escherichia coli* and *Staphylococcus aureus*. Together, these findings highlight the potential of essential oil-based strategies, with or without nanomaterial functionalization, to combat biofilm-associated challenges in food processing and clinical environments.

The *E. coli* strains were more sensitive to the ASO and PSO NEs, with an MIC of 1.06 μg/mL. The ASO NE showed the same MIC (1.06 μg/mL) against *B. oceanisediminis* strains, while the PSO NE required a higher MIC (4.25 μg/mL). *B. thuringiensis* strains showed MICs of 2.13 (ASO) and 4.25 μg/mL (PSO). Supporting these findings, studies on avocado ethanolic extracts demonstrated bactericidal activity against various pathogens: one attributed activity against *P. mirabilis*, *P. aeruginosa*, *S. aureus*, and *A. niger*, to alkaloids and saponins [55]; another study found that Fuerte, Hass, and Shepard avocado seeds inhibited *Clostridium sporogenes*, *S. enteritidis*, *P. aeruginosa*, and *Citrobacter freundii* (MICs 7.8 to 15.6 μg/mL, and bactericidal effects at 19.5 μg/mL), attributing this activity to polyphenolic compounds, including catechins, flavones, and tannins [56].

Pumpkin seed extracts have demonstrated antibacterial activity against *S. aureus* and *S. typhi* (0.6–0.7 mm) due to saponins and tannins [23], as well as against *B. subtilis* (1 mg/mL), *E. coli* (2 mg/mL), and *Klebsiella* (3 mg/mL) [106]. These compounds are thought to disrupt the phospholipid chain of the cell wall [107]. The presence of β-sitosterol may also contribute to the antimicrobial activity of ASO; although individually weak, it exhibits significant synergism against both gram-positive and gram-negative pathogens by inhibiting peptidoglycan biosynthesis and cell wall formation [108,109,110]. On the other hand, PSO exhibited high activity against *Klebsiella pneumoniae* and *Acinetobacter baumannii* at 16 μg/mL [111]. Overall, the antibacterial activity of SOs is attributed to a synergy of compounds including phytosterols, triterpenoids, phenolic compounds and derivatives, coumarins, unsaturated fatty acids, flavonoids, carotenoids, saponins, squalene, pyrazine, and minerals [15].

#### 3.7.2. Antibacterial Activity of Oil Mixtures

The results showed that NEs formulated with a mixture of oils were, in general, more effective than those with single oils, although few components show true synergist effects, most exhibited antagonistic and additive effects [112]. As indicated in Table 9, the Mix EBM, Mix AP, and Mix EBM + AP had an additive effect on *E. coli* strains. Against *B. oceanisediminis* all mixtures were additive except for Mix EBM, which showed synergy. For *B. thuringiensis* both Mix AP and Mix EBM + AP were additive. Regarding *S. enterica* strains, no clear interactions were determined because basil essential oil alone had the lowest MIC (2.13 µL/mL), whereas the mixtures containing it at lower percentages, such as the 50% in Mix EBM (MIC 4.25 µL/mL) and the 25% in Mix EBM + AP (10 µL/mL), were less effective. This indicates that the MIC in this case depends on basil oil and the type of interaction among the mixture components.

The antibacterial activity of essential oils (EOs) arises not merely from individual major compounds but from synergistic interactions among multiple components [113]. A key mechanism involves membrane sensitization: minor components facilitate the action of major ones. For example, p-cymene, a minor component, increases bacterial membrane fluidity, thereby enhancing membrane disruption caused by the major compound carvacrol [114]. Similarly, minor components such as 1,8-cineole and camphor have been shown to potentiate the activity of eugenol in clove and cinnamon oil vapors [115]. Advanced chemometric analyses further confirm that minor oxygenated sesquiterpenes can be primarily responsible for the overall antibacterial effect [116,117]. Beyond binary mixtures, triple combinations have demonstrated enhanced efficacy: a mixture of carvacrol, thymol, and eugenol was found to be effective against *L. innocua* [100], and triple combinations of Shiraz, thyme, clove, and cinnamon EOs showed synergistic effects (FIC_+_ ≤ 0.5) against *P. fluorescens* [118]. In the present study, mixtures (Mix EBM, Mix AP) showed lower MICs than individual components against *E. coli* and *B. oceanisediminis*, while the triple Mix EBM+AP exhibited MICs equal to single components, indicating additive effects attributed to acids, phenols, sterols, and tocopherols and confirming that mixtures were more effective (Table 9).

It is also important to highlight that the Mix AP and Mix EBM + AP NEs in the physical characterization conducted at 0 days of storage showed the smallest droplet sizes compared with the others, at 10.76 nm and 18.23 nm, respectively (see Figure 1A). Pearson’s correlation analysis showed a moderate positive relationship with *E. coli* (0.6) and *B. thuringiensis* (0.6), and strong positive relationship with *B. oceanisediminis* (0.9). In contrast, a perfect negative correlation (−1) was found for *S. enterica*, indicating that basil oil concentration in the Mix EBM and Mix EBM+AP NEs influenced MICs more than droplet size variable.

#### 3.7.3. Inactivation Kinetics

**Figure 2:**
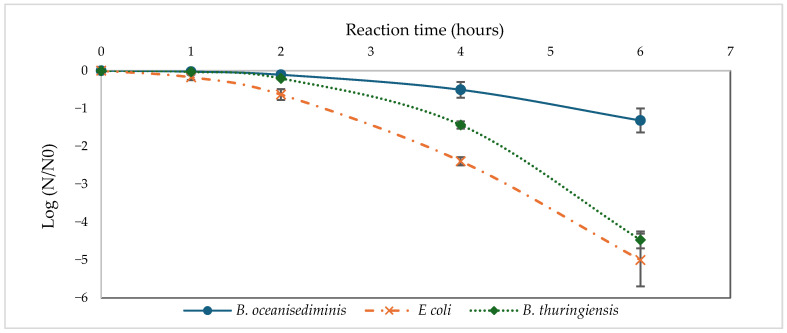
Survival fraction of *E. coli*, *B. oceanisediminis*, and *B. thuringiensis* after 6 h of reaction with the Mix AP nanoemulsion. The graphs of log (N/N_0_) vs. reaction time (hours) correspond to data fitted to a Weibull equation. Values are expressed as mean ± standard deviation.

Figure 2 depicts the inactivation kinetics of NE Mix AP against the strains *B. oceanisediminis* (TJC1), *E. coli* (TJC7), and *B. thuringiensis* (TJC2), selected for their high population density. A Weibull equation was fitted to the experimental data of the survival curves, revealing strain-dependent behavior. The Weibull model provided the best fit for *Bacillus* strains, (R^2^ > 0.96) whereas the fit for *E. coli* was weaker (R^2^: 0.81), indicating greater data dispersion and possible influence of other factors during NE treatment (Table 10).

This is also reflected in the precision factor *Af*, which indicated the difference between the values predicted by the model and the experimental values. *E. coli* presented the highest value (Table 10), indicating that the model predictions differed from the observations by 73%, a value higher than that obtained for *B. oceanisediminis* and *B. thuringiensis.* Therefore, the predictive capacity of the model is more limited and could be conditioned by the sensitivity of the bacteria to the individual and synergistic components of the nanoemulsion [46].

The shape factor (the Weibull model’s α parameter) exceeded 1.0 (Table 10) for all three strains evaluated, indicating a downward concavity, an initial resistance or tolerance phase, followed by accelerated inactivation due to the accumulated damage. This initial resistance phase may be related to the nanoemulsion’s zeta potential (−25.20 mV), which indicates moderate droplet repulsion, preventing aggregation and maintaining the nanoemulsion stability during storage. However, it can also cause repulsions with the cell walls of bacteria that possess a predominantly negative surface charge.

The scaling factor (β parameter), representing the time to the first logarithmic reduction in the bacterial population, varied by strain: the *E. coli* strain (gram-negative) required the shortest time (β = 1.62 h) while the two gram-positive *Bacillus* strains needed longer exposure (Table 10). Bactericidal activity against *E. coli* occurred at around 6 h of exposure time with NE with no cell growth observed after 48 h of incubation followed the treatment.

This finding align with Anwar et al. [119], who reported that non-polar extracts of *K. tomentosa* containing β-sitosterol exhibited an antimicrobial activity against *K. pneumonieae* (gram-negative) that was superior to that found against *S. aureus* (gram-positive). They noted that the thick peptidoglycan layer of gram-positive bacteria is more difficult to penetrate than the thin peptidoglycan structure of gram-negative bacteria, and that the outer membrane, composed of lipopolysaccharides, lipoproteins, and lipids, can be easily dissolved by emulsifiers or by active compounds in oils such as β-sitosterol [109]. Similarly, Amin et al. [120] also studied the antimicrobial activity of extracted pumpkin seed oil against strains of *E. coli*, finding activity similar to that of the gentamicin control.

Despite the strong stability and in vitro efficacy of the developed NEs, this study is limited by the lack of testing in a real matrix under commercial storage conditions. Furthermore, the effects of volatile oils on the sensory and nutritional quality of the fish were not evaluated.

## 4. Conclusions

This study demonstrated that most of the nanoemulsion formulations exhibited thermodynamic and physical stability for up to 90 days of storage, with greater antioxidant activity compared with that of the pure oils. Antibacterial evaluation showed MICs of 0.53–10 µL/mL against *E. coli* and *B. oceanisediminis*, while avocado, pumpkin, Mix AP, and Mix EBM+AP NEs inhibited *B. thuringiensis* (MIC: 2.13–4.25 µL/mL). Only basil oil NEs were active against *S. enterica* (MIC: 2.13–10 µL/mL). The FICI revealed additive and synergistic effects of the essential and seed oil compounds. Their potential incorporation into food systems could improve food safety by inhibiting pathogenic microorganisms that cause the spoilage of fishery products, thereby extending shelf life and reducing the risk of foodborne illnesses. Future studies should focus on optimizing their stability and efficacy under a real matrix at storage conditions as well as to assess the impact on sensorial and nutritional quality of the fish product.

## Figures and Tables

**Table 1:** Moisture and yield of oils by extraction method.

Raw Material	Extraction Technique	Moisture (%Wb)	Extraction Yield (%)
Mandarin peels	HD	69.43 ± 0.06	0.75 ± 0.04
Basil leaves	HD	84.33 ± 0.08	0.69 ± 0.03
Eucalyptus leaves	HD	85.17 ± 0.06	0.85 ± 0.04
Avocado seeds	SCF	13.30 ± 0.06	2.72 ± 0.03
Pumpkin seeds	SCF	11.67 ± 0.06	24.48 ± 0.03

HD: hydrodistillation. SCF: supercritical fluids. Wb: wet base. Values are the mean ± standard deviation of three samples per analysis.

**Table 2:** Quality indexes of essential and seed oils (AOAC standard).

Oils	Refraction	Density	Acidity	Peroxides	Iodine	Saponification
		g/mL	mg KOH/g Oil	mEq Active O_2_/kg Oil	g I_2_/100 g Oil	mg KOH/g Oil
Avocado	1.47 ± 0.01 ^a^	0.91 ± 0.03 ^bc^	1.55 ± 0.06 ^a^	3.49 ± 0.16 ^a^	87.80 ± 1.35 ^a^	187.30 ± 0.70 ^b^
Pumpkin	1.47 ± 0.02 ^a^	0.83 ± 0.02 ^a^	2.11 ± 0.06 ^b^	4.20 ± 0.17 ^b^	97.64 ± 1.13 ^b^	177.77 ± 0.75 ^a^
Mandarin	1.47 ± 0.02 ^a^	0.86 ± 0.02 ^ab^	-	-	-	-
Eucalyptus	1.46 ± 0.01 ^a^	0.92 ± 0.02 ^bc^	-	-	-	-
Basil	1.47 ± 0.03 ^a^	0.96 ± 0.02 ^c^	-	-	-	-

-: not applicable. Values are the mean ± standard deviation of three samples per analysis. Different lowercase letters with superscripts between rows indicate significant differences according to Tukey’s post hoc test (HSD) at *p* < 0.05.

**Table 3:** Chemical composition of essential oils (eucalyptus, mandarin, basil) obtained by GC–MS.

Mandarin Compounds	RA%	IRL	Eucalyptus Compounds	RA%	IRL	Basil Compounds	RA%	IRL
α-tujene	0.22 ± 0.12	926	α-tujene	0.15 ± 0.08	927	etil isovalerate	0.11 ± 0.03	853
α-pinene	0.89 ± 0.21	933	α-pinene	22.81 ± 0.33	936	α-pinene	1.48 ± 0.10	932
β-pinene	0.90 ± 0.13	976	β-pinene	1.53 ± 0.12	976	canfene	2.50 ± 0.18	947
β-mircene	2.79 ± 0.52	992	β-mircene	1.85 ± 0.11	992	sabinene	0.50 ± 0.03	973
octanal	3.52 ± 0.65	1005	α-felandrene	0.23 ± 0.02	1004	β-pinene	2.66 ± 0.24	976
2-carene	0.26 ± 0.09	1019	eucaliptol	57.85 ± 0.32	1037	β-mircene	0.94 ± 0.02	991
limonene	70.88 ± 0.53	1043	γ-terpinene	0.75 ± 0.07	1061	1-octan-3-ol	0.20 ± 0.10	1003
cis-β-ocimene	0.37 ± 0.04	1052	α-terpinolene	0.28 ± 0.05	1090	2-carene	0.13 ± 0.01	1017
γ-terpinene	7.74 ± 0.11	1064	3-metilbutil pentanoate	0.24 ± 0.01	1105	eucaliptol	24.12 ± 0.97	1036
1-octanol	0.69 ± 0.05	1078	p-menten-8-ol	0.11 ± 0.01	1175	*trans*-β-ocimene	0.34 ± 0.02	1039
α-terpinolene	0.44 ± 0.08	1091	4-terpineol	0.46 ± 0.12	1184	*cis*-β-ocimene	2.75 ± 0.37	1050
linalool	6.69 ± 0.39	1107	α-terpineol	1.27 ± 0.13	1199	γ-terpinene	0.27 ± 0.04	1060
citronela	0.22 ± 0.04	1155	geraniol	0.73 ± 0.07	1260	*trans*-sabinene hydrate	1.25 ± 0.05	1072
terpinen-4-ol	0.33 ± 0.07	1185	α-terpinil acetate	3.72 ± 0.17	1355	α-terpinolene	0.41 ± 0.03	1089
α-terpineol	0.43 ± 0.04	1200	geranil acetate	0.60 ± 0.22	1383	cis-sabinene hydrate	0.21 ± 0.06	1105
decanal	0.43 ± 0.19	1207	α-gurgujene	0.38 ± 0.18	1419	linalool	0.25 ± 0.01	1109
cis-geraniol	0.18 ± 0.01	1234	aromadendrene	1.49 ± 0.14	1451	camphor	24.61 ± 1.03	1156
cis-citral	0.21 ± 0.05	1245	Aloaromadendrene	0.47 ± 0.11	1473	exo-methyl-camfenilol	0.08 ± 0.02	1158
2-decenal	0.10 ± 0.01	1265	3-metil-2-fenilethylbutanoate	0.28 ± 0.08	1498	4-terpineol	0.69 ± 0.08	1176
e-citral	0.35 ± 0.09	1275	ledene	0.40 ± 0.10	1507	α-terpineol	0.46 ± 0.01	1184
Methyl benzoate	0.44 ± 0.13	1283	ledol	0.25 ± 0.06	1578	p-allilanisol	5.96 ± 0.03	1204
Thymol	1.25 ± 0.52	1313	globulal	0.22 ± 0.11	1586	estragol	4.95 ± 0.41	1207
farnesene	0.13 ± 0.03	1500	espatulenol	0.10 ± 0.03	1597	nerol	0.11 ± 0.01	1234
Total	99.46		viridiflorol	1.60 ± 0.24	1604	chavicol	2.43 ± 0.17	1282
			humulene epoxide	0.55 ± 0.02	1613	eugenol	4.22 ± 0.35	1371
			β-eudesmol	0.20 ± 0.11	1622	α-copaene	0.49 ± 0.03	1383
			rosifoliol	0.30 ± 0.03	1641	β-bourbonene	0.32 ± 0.01	1393
			γ-eudesmol	0.16 ± 0.09	1650	β-cubebene	0.25 ± 0.03	1396
			α-eudesmol	0.58 ± 0.07	1673	*trans*-caryophyllene	2.47 ± 0.04	1431
			Total	99.56		α-bergamotene	1.96 ± 0.01	1442
						β-sesquifelandrene	0.23 ± 0.02	1448
						*trans*-β-farnesene	0.17 ± 0.02	1458
						α-humulene	0.23 ± 0.02	1466
						germacrene D	3.51 ± 0.04	1494
						β-bisabolene	2.62 ± 0.03	1515
						α-copaene	0.15 ± 0.02	1532
						α-bisabolene	5.59 ± 0.38	1550
						viridiflorol	0.27 ± 0.01	1600
						Total	99.89	

RA%: relative abundance in percentage; IRL: relative retention index, calculated with DB-5MS column. Values are the mean ± standard deviation of three samples per analysis.

**Table 4:** Fatty acid profile of avocado and pumpkin seed oils.

Fatty Acid	Avocado Seed Oil		Pumpkin Seed Oil	
	TR	RA%	TR	RA%
Tridecanoic (13:0)	10.74	0.31 ± 0.02	ND	-
Myristic (14:0)	11.27	0.55 ± 0.06	ND	-
Palmitic (16:0)	12.43	29.70 ± 2.37	12.42	22.03 ± 0.26
Palmitoleic (16:1)	12.60	1.94 ± 0.57	ND	-
Estearic (18:0)	14.00	15.01 ± 0.68	14.00	12.16 ± 0.32
Oleic (18:1)	14.21	23.80 ± 5.39	14.19	24.61 ± 0.43
Linoleic (18:2)	14.67	28.16 ± 2.05	14.67	40.37 ± 0.45
Eicosanoic (20:0)	16.31	0.53 ± 0.10	16.30	0.53 ± 0.00
Heptadecanoic (17:0)	ND	-	13.11	0.09 ± 0.04
Linolenic (18:3)	ND	-	15.35	0.23 ± 0.02

TR: average retention time in minutes, with no standard deviations as they were minimally appreciable. RA%: mean ± standard deviation of the relative abundance percentage with three samples analyzed. ND: not detected.

**Table 5:** Sterols and tocopherols present in avocado and pumpkin seed oils.

Compounds	Avocado Seed Oil		Pumpkin Seed Oil	
	RA%	[mg/100 g]	RA%	[mg/100 g]
α-tocopherol	0.32 ± 0.04	9 ± 0.10	ND	-
γ-tocopherol	ND	-	0.10 ± 0.03	30 ± 0.10
Campesterol	0.11 ± 0.04	3 ± 0.10	ND	-
Stigmasterol	0.42 ± 0.03	120 ± 0.30	0.36 ± 0.11	100 ± 0.30
β-sitosterol	1.19 ± 0.11	50 ± 0.30	0.17 ± 0.07	34 ± 0.20

RA%: mean ± standard deviation of the relative abundance percentage with three samples analyzed. ND: not detected.

**Table 6:** Thermodynamic stability of nanoemulsions at 4 °C over 180 days.

Nanoemulsions	Centrifugation Stability					Heating and Cooling					Freeze–Thaw Stress				
						Days									
	0	15	30	90	180	0	15	30	90	180	0	15	30	90	180
Eucalyptus	N	N	N	N	C	N	N	N	N	C	N	N	N	N	C
Basil	N	N	N	N	C	N	N	N	N	C	N	N	N	N	C
Mandarin	N	N	N	N	N	N	N	N	N	C	N	N	N	N	C
Mix EBM	N	N	N	N	N	N	N	N	N	N	N	N	N	N	N
Avocado	N	N	N	N	N	N	N	N	N	N	N	N	N	N	N
Pumpkin	N	N	N	N	C	N	N	N	N	C	N	N	N	N	C
Mix AP	N	N	N	N	N	N	N	N	N	C	N	N	N	N	C
Mix EBM+AP	N	N	N	N	N	N	N	N	N	N	N	N	N	N	N

C: formation of cremated. N: normal.

**Table 7:** Antioxidant activity and total phenolic content in nanoemulsions and pure oils.

Methods		ABTS^•+^	DPPH	TPC
Nanoemulsions	Eucalyptus	1.63 ± 0.08 ^aB^	1.86 ± 0.07 ^aB^	0.15 ± 0.03 ^aA^
	Pumpkin	4.29 ± 0.08 ^cJ^	4.19 ± 0.06 ^dJ^	0.28 ± 0.04 ^bA^
	Basil	5.38 ± 0.08 ^dD^	4.73 ± 0.07 ^eD^	0.31 ± 0.03 ^bA^
	Avocado	69.36 ± 0.10 ^gH^	40.03 ± 0.10 ^hH^	2.67 ± 0.03 ^eA^
	Mandarin	1.85 ± 0.06 ^aF^	2.24 ± 0.08 ^bF^	0.25 ± 0.01 ^bA^
	Mix AP	51.03 ± 0.10 ^f^	28.21 ± 0.10 ^g^	1.93 ± 0.04 ^d^
	Mix EBM	3.57 ± 0.10 ^b^	3.40 ± 0.07 ^c^	0.27 ± 0.02 ^b^
	Mix EBM + AP	18.14 ± 0.10 ^e^	11.53 ± 0.10 ^f^	1.11 ± 0.03 ^c^
Pure oils	Eucalyptus	0.31 ± 0.06 ^aA^	0.38 ± 0.03 ^aA^	0.15 ± 0.02 ^aA^
	Pumpkin	1.01 ± 0.07 ^bI^	1.07 ± 0.06 ^cI^	0.28 ± 0.02 ^bA^
	Basil	2.49 ± 0.13 ^cC^	1.24 ± 0.05 ^dC^	0.31 ± 0.02 ^bA^
	Avocado	66.69 ± 0.10 ^dG^	37.17 ± 0.04 ^eG^	2.67 ± 0.02 ^cA^
	Mandarin	0.34 ± 0.08 ^aE^	0.74 ± 0.05 ^bE^	0.25 ± 0.03 ^bA^

Values are the mean ± standard deviation of three samples per analysis. Different lowercase letters with superscripts between rows in each of the nanoemulsions and pure oils group indicate significant differences according to Tukey’s post hoc test (HSD) at *p* < 0.05. The superscripted capital letters between the rows indicate significant differences between nanoemulsions and pure oils according to Tukey’s post hoc test (HSD) at *p* < 0.05. ABTS and DPPH in µmol TE/mL oil. TPC in mg GAE/mL oil.

**Table 8:** Antibacterial activity of nanoemulsions and oils.

Specie		*E. coli*			*B. oceanisediminis*			*B. thuringiensis*			*K. variicola*			*S. enterica*		
Strains TJC		3	7	10	1	18	8	2	20	25	15	19	11	5	13	21
Nanoemulsions	Eucalyptus	10	10	10	ND	ND	ND	ND	ND	ND	ND	ND	ND	ND	ND	ND
	Basil	2.13	2.13	2.13	10	10	10	2.13	2.13	2.13	ND	ND	ND	2.13	2.13	2.13
	Mandarin	4.25	4.25	4.25	10	10	10	ND	ND	ND	ND	ND	ND	ND	ND	ND
	Mix EBM	1.06	1.06	1.06	2.13	2.13	2.13	ND	ND	ND	ND	ND	ND	4.25	4.25	4.25
	Avocado	1.06	1.06	1.06	1.06	1.06	1.06	2.13	2.13	2.13	ND	ND	ND	ND	ND	ND
	Pumpkin	1.06	1.06	1.06	4.25	4.25	4.25	4.25	4.25	4.25	ND	ND	ND	ND	ND	ND
	Mix AP	0.53	0.53	0.53	0.53	0.53	0.53	2.13	2.13	2.13	ND	ND	ND	ND	ND	ND
	Mix EBM+AP	0.53	0.53	0.53	0.53	0.53	0.53	2.13	2.13	2.13	ND	ND	ND	10	10	10
Pure oils	Eucalyptus	40	50	50	ND	ND	ND	ND	ND	ND	ND	ND	ND	ND	ND	ND
	Basil	20	30	30	50	50	50	30	30	30	ND	ND	ND	30	30	30
	Mandarin	30	40	40	50	50	50	ND	ND	ND	ND	ND	ND	ND	ND	ND
	Avocado	20	20	20	20	20	20	30	40	30	ND	ND	ND	ND	ND	ND
	Pumpkin	20	20	20	40	30	40	40	40	40	ND	ND	ND	ND	ND	ND

Strains TJC: *E. coli* (3-7-10), *B. oceanisediminis* (1-18-8), *B. thuringiensis* (2-20-25), *K. variicola* (15-19-11), *S. enterica* (5-13-21). ND: Not detected. For this study, MIC = MBC (μL/mL).

**Table 9:** Fractional inhibition concentration index (FICI) of nanoemulsion mixtures and their components.

Bacterial Strains	Nanoemulsion	Alone	Combination	FIC	FICI	Outcome
		MIC (µL/mL)				
*E. coli* (TJC3, TJC7, TJC7)	Eucalyptus	10.00 ± 0.00		0.11	0.85	Additive
	Basil	2.13 ± 0.00		0.50		
	Mandarin	4.25 ± 0.00		0.25		
	MIX EBM		1.06 ± 0.00			
	Avocado	1.06 ± 0.00		0.50	1.00	Additive
	Pumpkin	1.06 ± 0.00		0.50		
	Mix AP		0.53 ± 0.00			
	MIX EBM	1.06 ± 0.00		0.50	1.50	Additive
	Mix AP	0.53 ± 0.00		1.00		
	Mix EBM + AP		0.53 ± 0.00			
*B. oceanisediminis* (TJC1, TJC18, TJC8)	Basil	10.00 ± 0.00		0.21	0.43	Synergistic
	Mandarin	10.00 ± 0.00		0.21		
	MIX EBM		2.13 ± 0.00			
	Avocado	1.06 ± 0.00		0.50	0.62	Additive
	Pumpkin	4.25 ± 0.00		0.12		
	Mix AP		0.53 ± 0.00			
	MIX EBM	2.13 ± 0.00		0.25	1.25	Additive
	Mix AP	0.53 ± 0.00		1.00		
	Mix EBM + AP		0.53 ± 0.00			
*B. thuringiensis* (TJC2, TJC20, TJC25)	Avocado	2.13 ± 0.00		1.00	1.50	Additive
	Pumpkin	4.25 ± 0.00		0.50		
	Mix AP		2.13 ± 0.00			
	MIX EBM	ND		0.00	1.00	Additive
	Mix AP	2.13 ± 0.00		1.00		
	Mix EBM + AP		2.13 ± 0.00			

The Σ FICI values are interpreted as follows: antagonistic if FICI > 4; additive if 0.5 < FICI < 4; synergistic if FICI ≤ 0.5. ND: Not detected. The MIC values correspond to the mean and standard deviation of the three strains for each type of bacteria.

**Table 10:** Estimated Weibull parameters describing bacterial inactivation with Mix AP nanoemulsion.

Strain	α	β	R^2^	A_f_
*B. oceanisediminis*	2.41 ± 0.47	3.70 ± 0.62	0.9995	1.42
*E. coli*	1.91 ± 0.31	1.62 ± 0.24	0.8156	1.73
*B. thuringiensis*	2.81 ± 0.12	2.61 ± 0.10	0.9603	1.21

Values are expressed as mean ± standard deviation, β is the scale factor; α is the shape factor; A_f_ is the accuracy factor.

## Data Availability

The original contributions presented in this study are included in the article/Supplementary Materials. Further inquiries can be directed to the corresponding authors.

## Supplementary Materials

The following supporting information can be downloaded at https://www.mdpi.com/article/10.3390/foods15132340/s1, Figure S1. Characterization of nanoemulsions performed with the dynamic light scattering method. Figure S2. Thermodynamic stability of nanoemulsions stored at 4 °C for 180 days. Table S1. Analysis of variance—ANOVA, Tukey’s post hoc tests (HSD) and Kruskal–Wallis for the statistical analysis of quality índexes, physical characterization and antioxidant activity of oils, and nanoemulsions.

## Abbreviations

The following abbreviations are used in this manuscript:

EOs	Essential oils
SOs	Seed oils
GC–MS	Gas chromatography–mass spectrometry
NEs	Nanoemulsions
NE	Nanoemulsion
FICI	Fractional inhibition concentration index
FIC	Fractional inhibition concentration
FBOs	Foodborne outbreaks
EFSA	European Food Safety Authority
AOAC	Association of Official Agricultural Chemists
O/W	Oil in water
*v*/*v*	Volumen de soluto a volumen de solución
EEO	Essential oil of eucalyptus
MEO	Essential oil of mandarin
BEO	Essential oil of basil
ASO	Oil of avocado seed
PSO	Oil of pumpkin seed
Mix EBM	Mix of essential oils of eucalyptus, basil, and mandarin
Mix AP	Mix of seed oils of avocado and pumpkin
Mix EBM + AP	Mix of essential and seed oils
PDI	Polydispersity index
Zp	Zeta potential
Ds	Droplet size
DPPH	2,2-diphenyl-1-picrylhydrazyl
ABTS^•+^	2,2′-azino-bis (3-ethylbenzothiazoline-6-sulfonic acid
TPC	Total phenolic content
GAE	Gallic acid equivalent
TSB	Tryptic soy broth
PBS	Phosphate-buffered saline
TTC	2,3,5-Triphenyl tetrazolium chloride
MBC	Minimum bactericidal concentration
MIC	Minimum inhibitory concentration
ANOVA	Analysis of variance
HD	Hydro distillation
SCFs	Super critical fluids
IRL	Relative retention index
ND	Not detected
*E. coli*	*Escherichia coli*
*B. oceanisediminis*	*Bacillus oceanisediminis*
*B. thuringiensis*	*Bacillus thuringiensis*
*K. variicola*	*Klebsiella variicola*
*S. enterica*	*Salmonella enterica*
*S. aureus*	*Staphylococcus aureus*
*L. monocytogenes*	*Listeria monocytogenes*
*P. mirabilis*	*Proteus mirabilis*
*P. aeruginosa*	*Pseudomonas aeruginosa*
*A. niger*	*Aspergillus niger*
*S. enteritidis*	*Salmonella. enteritidis*
*B. subtilis*	*Bacillus subtilis*
*K. pneumoniae*	*Klebsiella pneumoniae*
*L. innocua*	*Listeria innocua*
*K. tomentosa*	*Kalanchoe tomentosa*
*S. typhi*	*Salmonella typhi*
