# Supplementary material for: Antibacterial Activity of Nanoemulsions Prepared with Essential and Seed Oils Against Isolated Bacteria from Rainbow Trout (Oncorhynchus mykiss)
Source: Foods. 2026 Jul 2;15(13):2340. doi: 10.3390/foods15132340 (PMC13361915; doi:10.3390/foods15132340)
Supplement: Supplementary file 1 [file foods-15-02340-s001.zip › Figure S1.pdf]

			Size (d.nm):	% Intensity:	St Dev (d.nm):
Z-Average (d.nm):	169,4	Peak 1:	236,9	89,9	40,84
PdI:	0,524	Peak 2:	10,95	10,1	0,7966
Intercept:	0,910	Peak 3:	0,000	0,0	0,000

Result quality : Refer to quality report

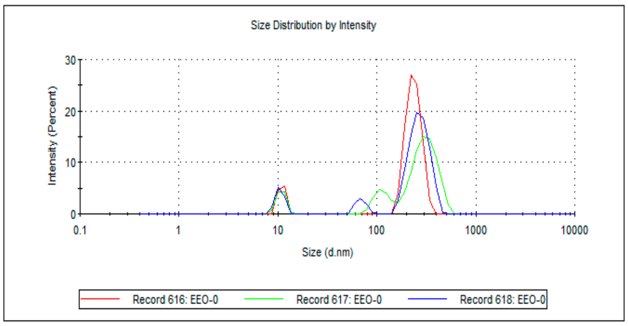

a

			Mean (mV)	Area (%)	St Dev (mV)
Zeta Potential (mV):	-22,0	Peak 1:	-22,0	100,0	4,04
Zeta Deviation (mV):	4,04	Peak 2:	0,00	0,0	0,00
Conductivity (mS/cm):	0,366	Peak 3:	0,00	0,0	0,00

Result quality : Good

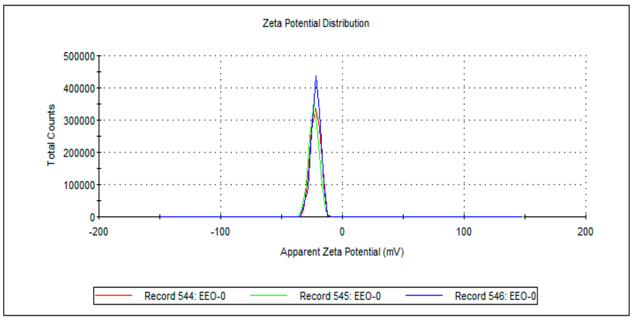

b

			Size (d.nm):	% Intensity:	St Dev (d.nm):
Z-Average (d.nm):	161,5	Peak 1:	181,9	75,2	20,05
PdI:	0,427	Peak 2:	9,831	24,8	0,9143
Intercept:	0,962	Peak 3:	0,000	0,0	0,000

Result quality : Refer to quality report

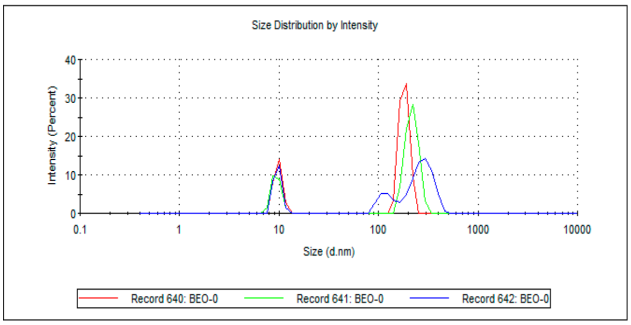

c

			Mean (mV)	Area (%)	St Dev (mV)
Zeta Potential (mV):	-17,2	Peak 1:	-19,7	79,4	5,94
Zeta Deviation (mV):	7,98	Peak 2:	-5,36	20,6	3,13
Conductivity (mS/cm):	0,0861	Peak 3:	0,00	0,0	0,00

Result quality : Good

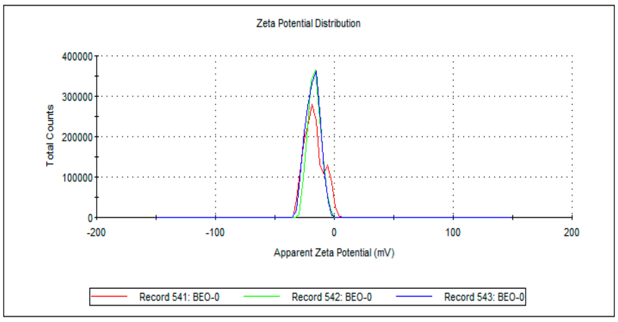

d

			Size (d.nm):	% Intensity:	St Dev (d.nm):
Z-Average (d.nm):	165,4	Peak 1:	250,8	67,9	36,27
PdI:	0,440	Peak 2:	10,75	24,3	1,723
Intercept:	0,948	Peak 3:	77,69	7,8	8,288

Result quality : Refer to quality report

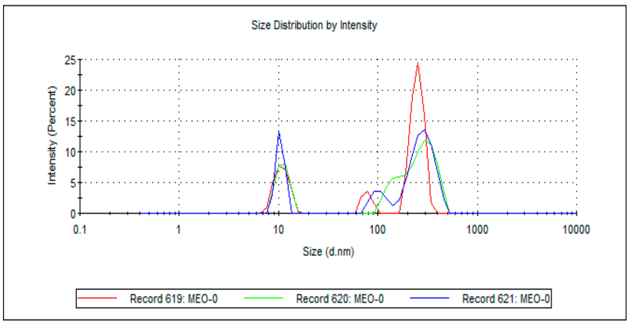

e

			Mean (mV)	Area (%)	St Dev (mV)
Zeta Potential (mV):	-24,6	Peak 1:	-25,0	99,1	8,51
Zeta Deviation (mV):	9,59	Peak 2:	23,4	0,9	1,95
Conductivity (mS/cm):	0,536	Peak 3:	0,00	0,0	0,00

Result quality : Good

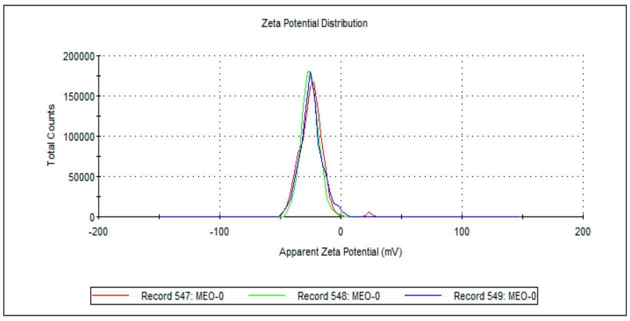

f

			Size (d.nm):	% Intensity:	St Dev (d.nm):
Z-Average (d.nm):	109,3	Peak 1:	9,360	67,8	0,7750
PdI:	0,269	Peak 2:	114,0	32,2	8,355
Intercept:	0,971	Peak 3:	0,000	0,0	0,000

Result quality : Refer to quality report

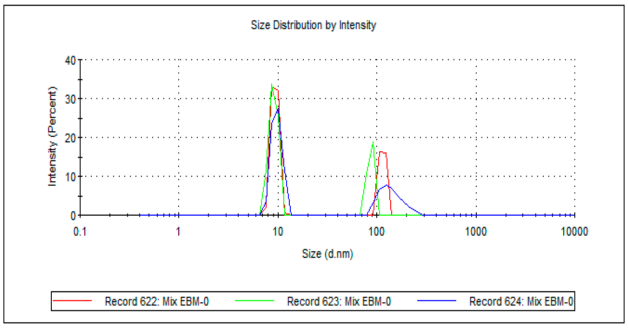

g

			Mean (mV)	Area (%)	St Dev (mV)
Zeta Potential (mV):	-17,7	Peak 1:	-17,7	100,0	3,89
Zeta Deviation (mV):	3,89	Peak 2:	0,00	0,0	0,00
Conductivity (mS/cm):	0,637	Peak 3:	0,00	0,0	0,00

Result quality : Good

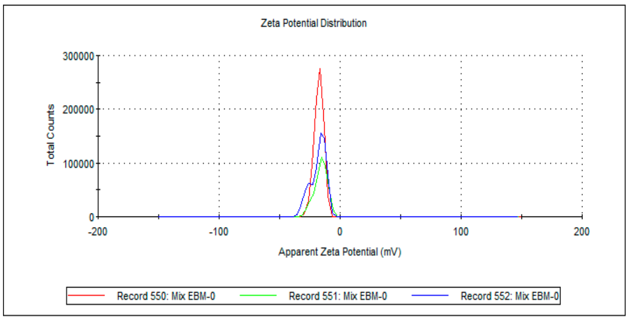

h

			Size (d.nm):	% Intensity:	St Dev (d.nm):
Z-Average (d.nm):	48,80	Peak 1:	154,6	55,7	55,22
PdI:	0,500	Peak 2:	29,04	35,1	8,131
Intercept:	0,719	Peak 3:	10,28	7,6	1,577

Result quality : Good

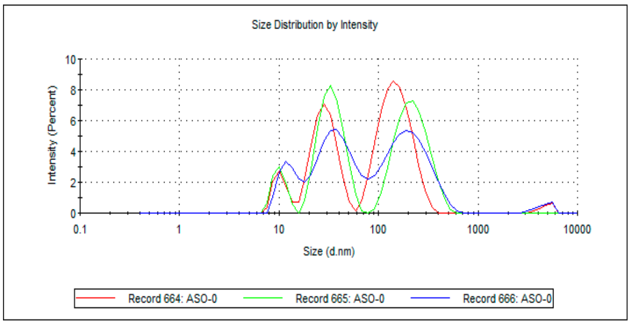

i

			Mean (mV)	Area (%)	St Dev (mV)
Zeta Potential (mV):	-20,7	Peak 1:	-20,7	100,0	4,72
Zeta Deviation (mV):	4,72	Peak 2:	0,00	0,0	0,00
Conductivity (mS/cm):	0,440	Peak 3:	0,00	0,0	0,00

Result quality : Good

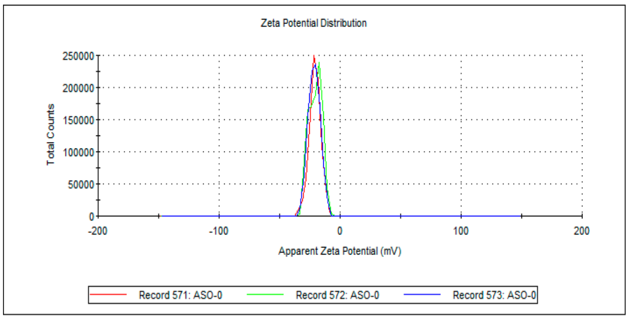

j

			Size (d.nm):	% Intensity:	St Dev (d.nm):
Z-Average (d.nm):	148,2	Peak 1:	184,6	89,7	53,87
PdI:	0,237	Peak 2:	59,50	10,3	10,26
Intercept:	0,922	Peak 3:	0,000	0,0	0,000

Result quality : Good

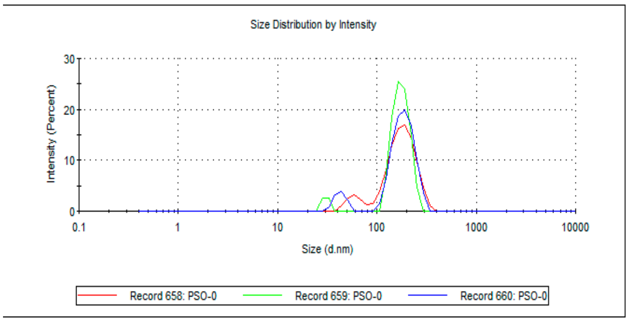

k

			Mean (mV)	Area (%)	St Dev (mV)
Zeta Potential (mV):	-26,9	Peak 1:	-26,9	100,0	8,35
Zeta Deviation (mV):	8,35	Peak 2:	0,00	0,0	0,00
Conductivity (mS/cm):	0,0391	Peak 3:	0,00	0,0	0,00

Result quality : Good

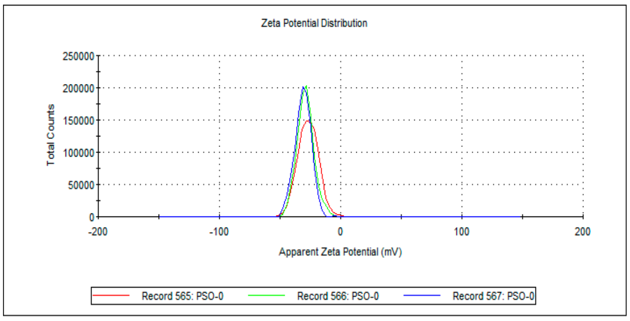

l

		Size (d.nm):	% Intensity:	St Dev (d.nm):
Z-Average (d.nm): 10,76	Peak 1:	9,676	64,4	3,833
PdI: 0,317	Peak 2:	31,16	35,6	10,82
Intercept: 0,678	Peak 3:	0,000	0,0	0,000

Result quality : Good

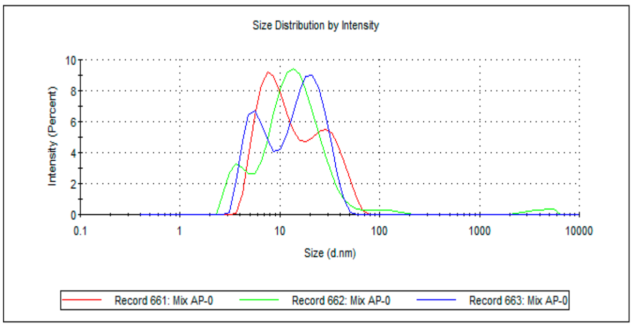

m

		Mean (mV)	Area (%)	St Dev (mV)
Zeta Potential (mV): -25,2	Peak 1:	-25,2	100,0	6,22
Zeta Deviation (mV): 6,22	Peak 2:	0,00	0,0	0,00
Conductivity (mS/cm): 0,352	Peak 3:	0,00	0,0	0,00

Result quality : Good

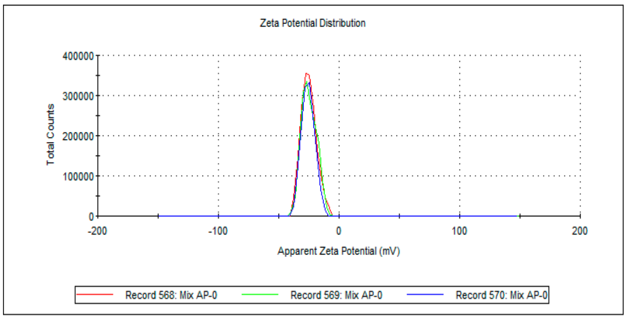

n

		Size (d.nm):	% Intensity:	St Dev (d.nm):
Z-Average (d.nm): 18,23	Peak 1:	14,09	80,4	3,105
PdI: 0,388	Peak 2:	99,99	19,6	25,19
Intercept: 0,934	Peak 3:	0,000	0,0	0,000

Result quality : Good

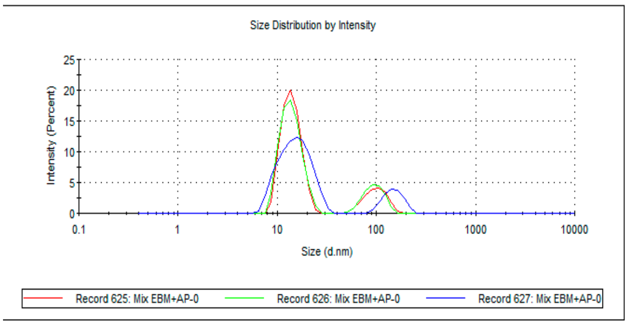

ñ

		Mean (mV)	Area (%)	St Dev (mV)
Zeta Potential (mV): -25,6	Peak 1:	-25,6	100,0	7,19
Zeta Deviation (mV): 7,19	Peak 2:	0,00	0,0	0,00
Conductivity (mS/cm): 0,554	Peak 3:	0,00	0,0	0,00

Result quality : Good

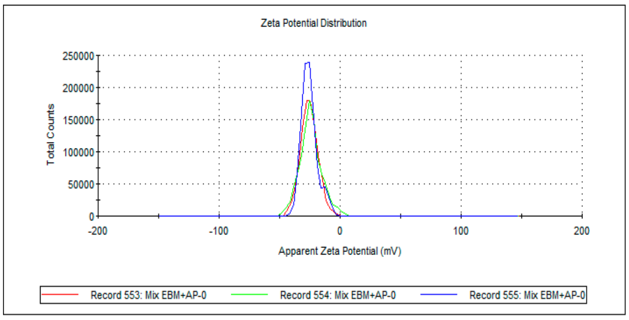

o

		Size (d.nm):	% Intensity:	St Dev (d.nm):
Z-Average (d.nm): 197,6	Peak 1:	292,2	80,1	44,81
PdI: 0,512	Peak 2:	11,97	11,7	1,104
Intercept: 0,917	Peak 3:	60,73	8,2	6,056

Result quality : Refer to quality report

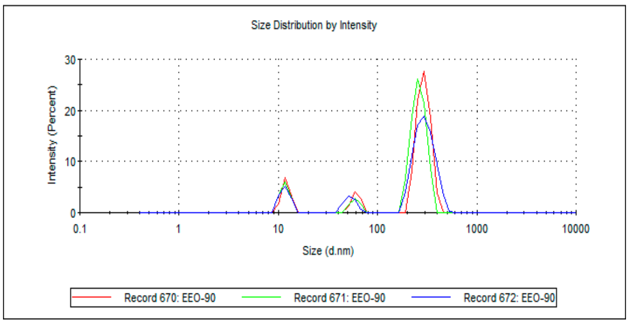

p

		Mean (mV)	Area (%)	St Dev (mV)
Zeta Potential (mV): -14,1	Peak 1:	-14,1	100,0	5,71
Zeta Deviation (mV): 5,71	Peak 2:	0,00	0,0	0,00
Conductivity (mS/cm): 0,0489	Peak 3:	0,00	0,0	0,00

Result quality : Good

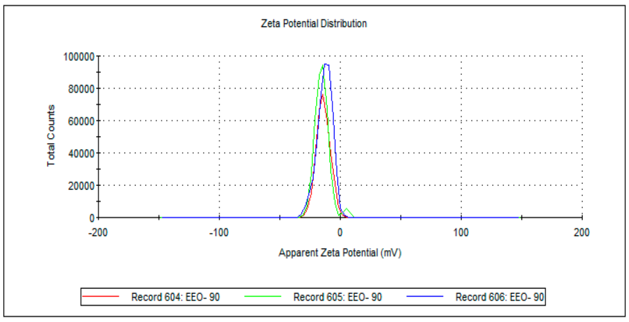

q

			Size (d.nm):	% Intensity:	St Dev (d.nm):
Z-Average (d.nm):	200,5	Peak 1:	175,6	44,9	19,84
PdI:	0,445	Peak 2:	77,14	20,2	8,163
Intercept:	0,980	Peak 3:	7,314	18,7	0,7903

Result quality : Refer to quality report

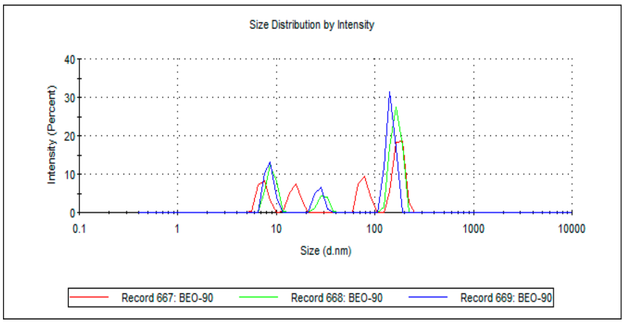

r

			Mean (mV)	Area (%)	St Dev (mV)
Zeta Potential (mV):	-13,8	Peak 1:	-13,8	100,0	5,42
Zeta Deviation (mV):	5,42	Peak 2:	0,00	0,0	0,00
Conductivity (mS/cm):	0,0399	Peak 3:	0,00	0,0	0,00

Result quality : Good

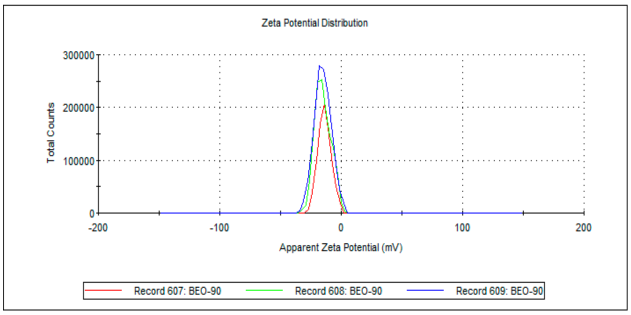

s

			Size (d.nm):	% Intensity:	St Dev (d.nm):
Z-Average (d.nm):	188,1	Peak 1:	10,58	54,9	1,986
PdI:	0,429	Peak 2:	175,0	45,1	12,80
Intercept:	0,981	Peak 3:	0,000	0,0	0,000

Result quality : Refer to quality report

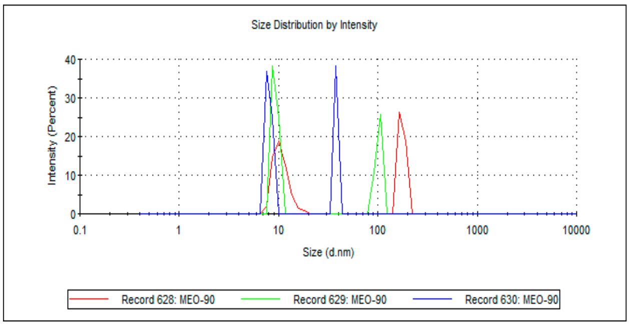

t

			Mean (mV)	Area (%)	St Dev (mV)
Zeta Potential (mV):	-10,5	Peak 1:	-10,5	100,0	4,10
Zeta Deviation (mV):	4,10	Peak 2:	0,00	0,0	0,00
Conductivity (mS/cm):	0,0286	Peak 3:	0,00	0,0	0,00

Result quality : Good

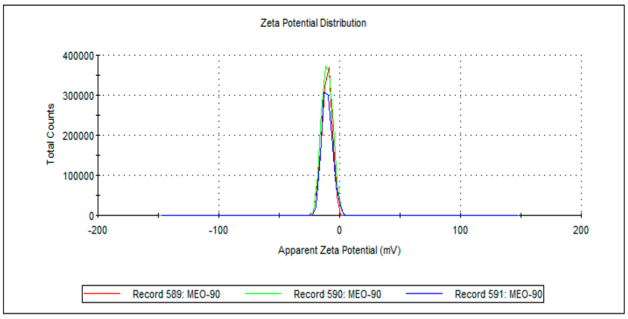

u

			Size (d.nm):	% Intensity:	St Dev (d.nm):
Z-Average (d.nm):	147,0	Peak 1:	223,5	88,3	40,04
PdI:	0,732	Peak 2:	11,17	11,7	1,059
Intercept:	0,929	Peak 3:	0,000	0,0	0,000

Result quality : Refer to quality report

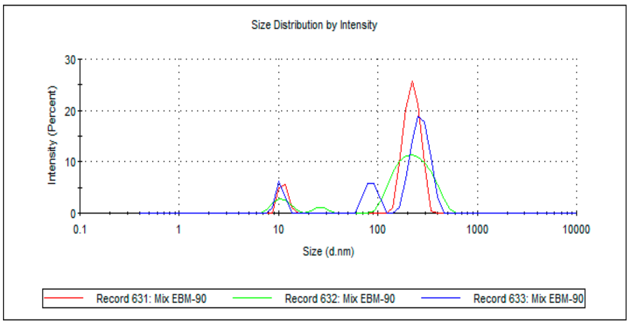

v

			Mean (mV)	Area (%)	St Dev (mV)
Zeta Potential (mV):	-13,9	Peak 1:	-14,3	98,6	7,16
Zeta Deviation (mV):	7,76	Peak 2:	13,8	1,4	3,30
Conductivity (mS/cm):	0,362	Peak 3:	0,00	0,0	0,00

Result quality : Good

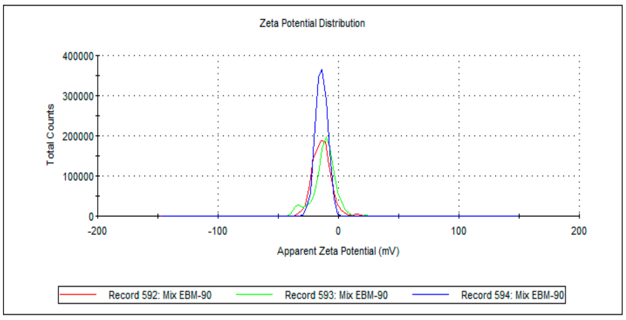

w

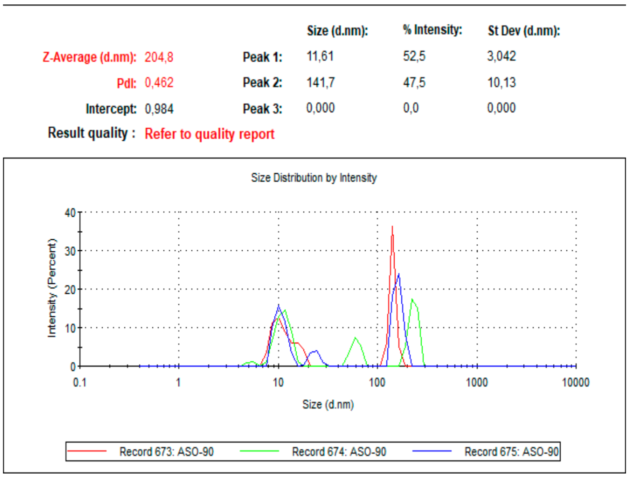
Size (d.nm): % Intensity: St Dev (d.nm):
Z-Average (d.nm): 204,8 Peak 1: 11,61 52,5 3,042
PdI: 0,462 Peak 2: 141,7 47,5 10,13
Intercept: 0,984 Peak 3: 0,000 0,0 0,000
Result quality : Refer to quality report
Size Distribution by Intensity
Intensity (Percent)
Size (d.nm)
Record 673: ASO-90 Record 674: ASO-90 Record 675: ASO-90

x

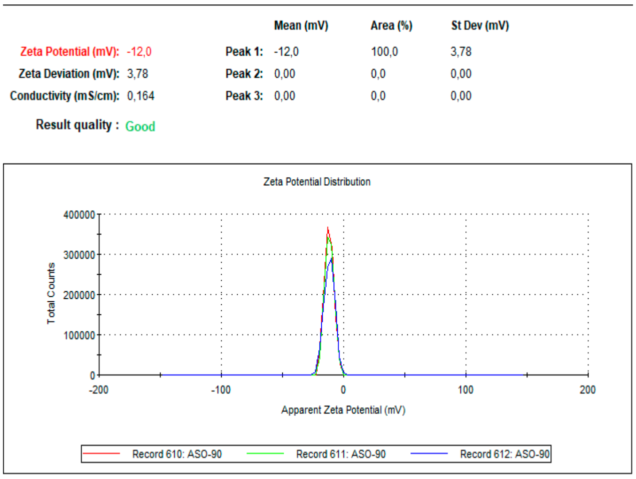
Mean (mV) Area (%) St Dev (mV)
Zeta Potential (mV): -12,0 Peak 1: -12,0 100,0 3,78
Zeta Deviation (mV): 3,78 Peak 2: 0,00 0,0 0,00
Conductivity (mS/cm): 0,164 Peak 3: 0,00 0,0 0,00
Result quality : Good
Zeta Potential Distribution
Total Counts
Apparent Zeta Potential (mV)
Record 610: ASO-90 Record 611: ASO-90 Record 612: ASO-90

y

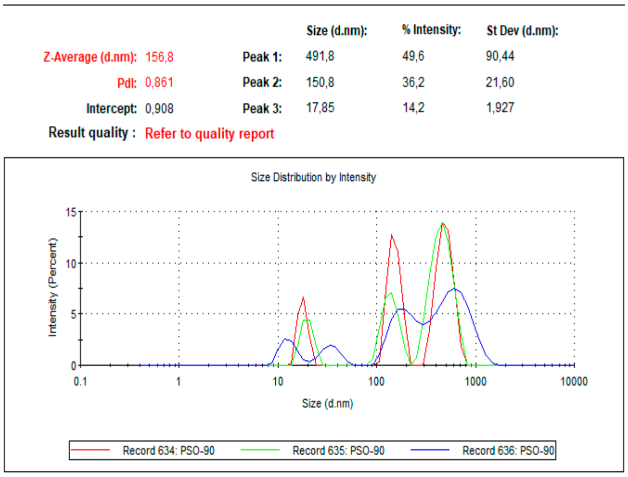
Size (d.nm): % Intensity: St Dev (d.nm):
Z-Average (d.nm): 156,8 Peak 1: 491,8 49,6 90,44
PdI: 0,861 Peak 2: 150,8 36,2 21,60
Intercept: 0,908 Peak 3: 17,85 14,2 1,927
Result quality : Refer to quality report
Size Distribution by Intensity
Intensity (Percent)
Size (d.nm)
Record 634: PSO-90 Record 635: PSO-90 Record 636: PSO-90

z

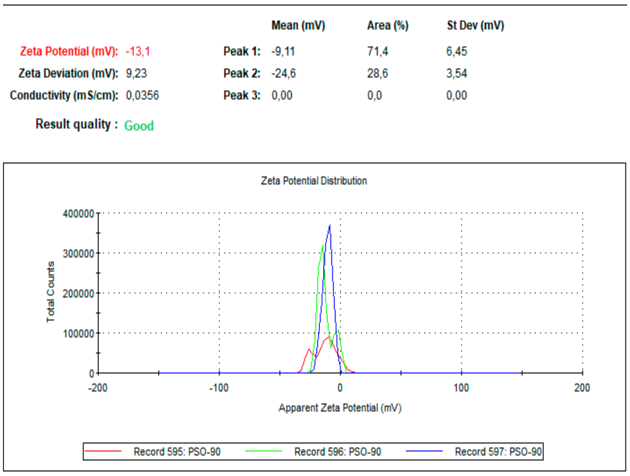
Mean (mV) Area (%) St Dev (mV)
Zeta Potential (mV): -13,1 Peak 1: -9,11 71,4 6,45
Zeta Deviation (mV): 9,23 Peak 2: -24,6 28,6 3,54
Conductivity (mS/cm): 0,0356 Peak 3: 0,00 0,0 0,00
Result quality : Good
Zeta Potential Distribution
Total Counts
Apparent Zeta Potential (mV)
Record 595: PSO-90 Record 596: PSO-90 Record 597: PSO-90

a′

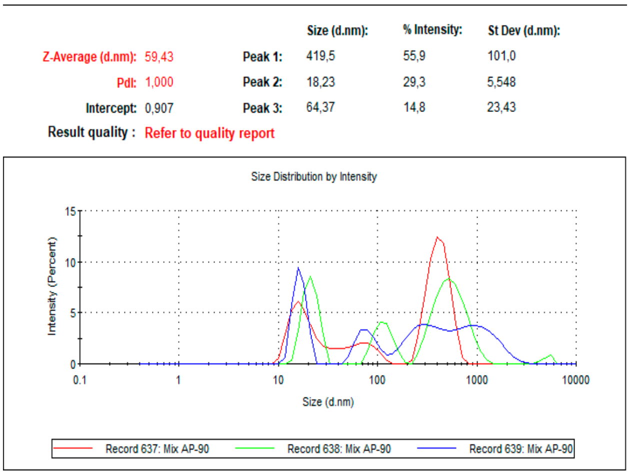
Size (d.nm): % Intensity: St Dev (d.nm):
Z-Average (d.nm): 59,43 Peak 1: 419,5 55,9 101,0
PdI: 1,000 Peak 2: 18,23 29,3 5,548
Intercept: 0,907 Peak 3: 64,37 14,8 23,43
Result quality : Refer to quality report
Size Distribution by Intensity
Intensity (Percent)
Size (d.nm)
Record 637: Mix AP-90 Record 638: Mix AP-90 Record 639: Mix AP-90

b′

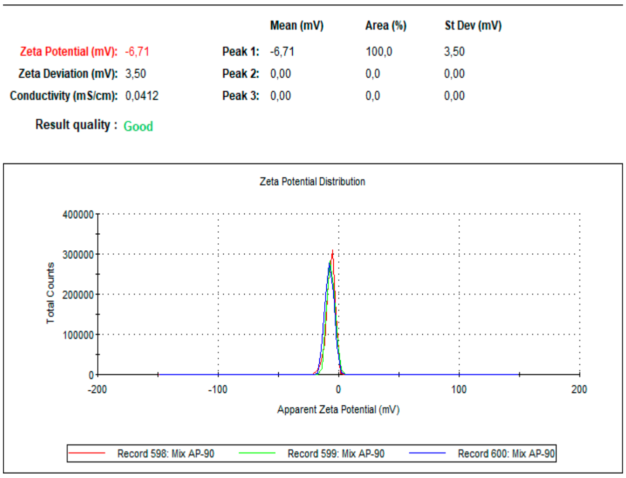
Mean (mV) Area (%) St Dev (mV)
Zeta Potential (mV): -6,71 Peak 1: -6,71 100,0 3,50
Zeta Deviation (mV): 3,50 Peak 2: 0,00 0,0 0,00
Conductivity (mS/cm): 0,0412 Peak 3: 0,00 0,0 0,00
Result quality : Good
Zeta Potential Distribution
Total Counts
Apparent Zeta Potential (mV)
Record 598: Mix AP-90 Record 599: Mix AP-90 Record 600: Mix AP-90

c′

			Size (d.nm):	% Intensity:	St Dev (d.nm):
Z-Average (d.nm):	158,8	Peak 1:	339,2	75,2	71,36
PdI:	0,661	Peak 2:	32,66	16,3	5,037
Intercept:	0,927	Peak 3:	11,41	8,5	1,366

Result quality : Refer to quality report

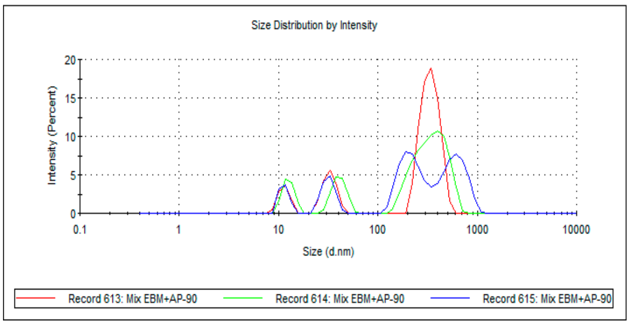

d´

			Mean (mV)	Area (%)	St Dev (mV)
Zeta Potential (mV):	-12,2	Peak 1:	-15,0	61,5	4,29
Zeta Deviation (mV):	5,79	Peak 2:	-6,78	38,5	2,41
Conductivity (mS/cm):	0,0349	Peak 3:	0,00	0,0	0,00

Result quality : Good

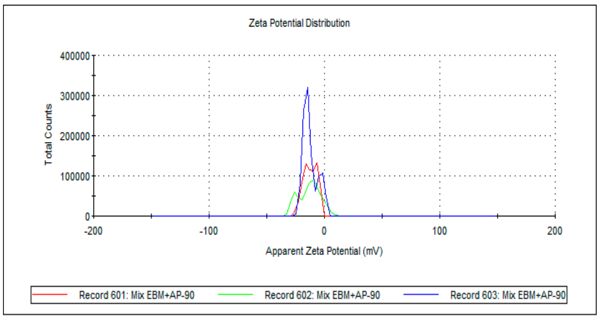

e´

			Size (d.nm):	% Intensity:	St Dev (d.nm):
Z-Average (d.nm):	1040	Peak 1:	97,87	84,0	7,187
PdI:	1,000	Peak 2:	7,463	16,0	0,2552
Intercept:	1,16	Peak 3:	0,000	0,0	0,000

Result quality : Refer to quality report

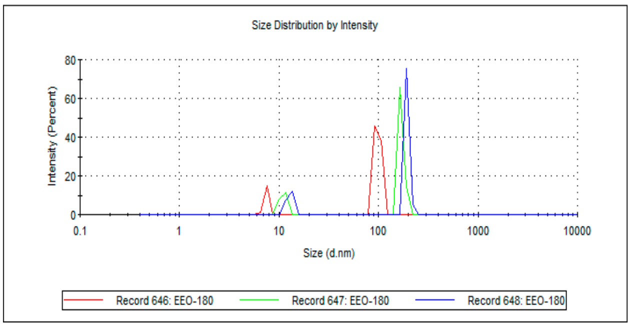

f´

			Mean (mV)	Area (%)	St Dev (mV)
Zeta Potential (mV):	-11,2	Peak 1:	-6,87	67,4	9,36
Zeta Deviation (mV):	69,9	Peak 2:	38,8	7,5	10,5
Conductivity (mS/cm):	0,371	Peak 3:	-36,2	3,7	3,52

Result quality : See result quality report

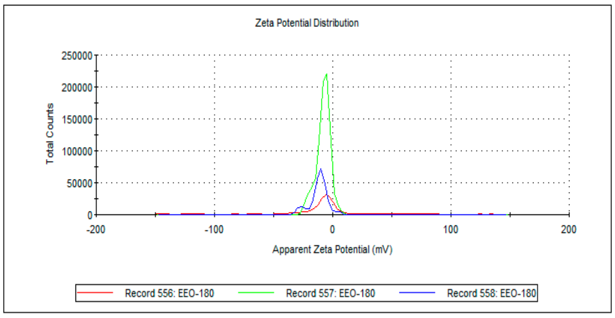

g´

			Size (d.nm):	% Intensity:	St Dev (d.nm):
Z-Average (d.nm):	1186	Peak 1:	155,8	64,7	10,84
PdI:	1,000	Peak 2:	9,708	35,3	0,6216
Intercept:	1,24	Peak 3:	0,000	0,0	0,000

Result quality : Refer to quality report

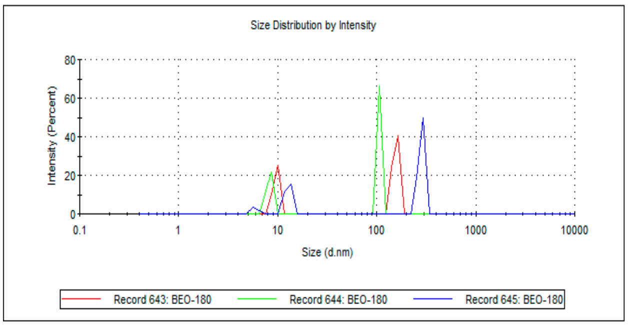

h´

			Mean (mV)	Area (%)	St Dev (mV)
Zeta Potential (mV):	-17,5	Peak 1:	-12,1	50,8	11,2
Zeta Deviation (mV):	59,0	Peak 2:	-38,3	12,2	6,12
Conductivity (mS/cm):	0,0975	Peak 3:	-57,0	6,1	5,29

Result quality : See result quality report

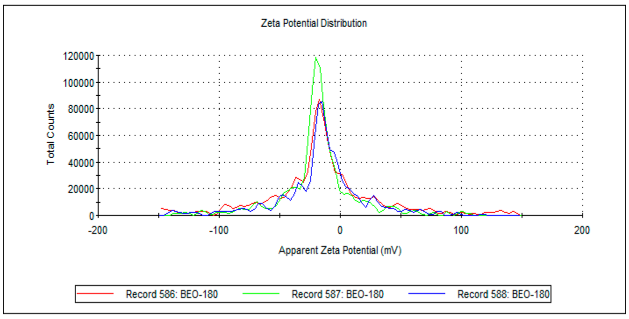

i´

			Size (d.nm):	% Intensity:	St Dev (d.nm):
Z-Average (d.nm):	855,4	Peak 1:	159,0	43,1	9,430
PdI:	0,999	Peak 2:	1106	42,1	0,000
Intercept:	1,36	Peak 3:	10,58	7,6	0,7327

Result quality : Refer to quality report

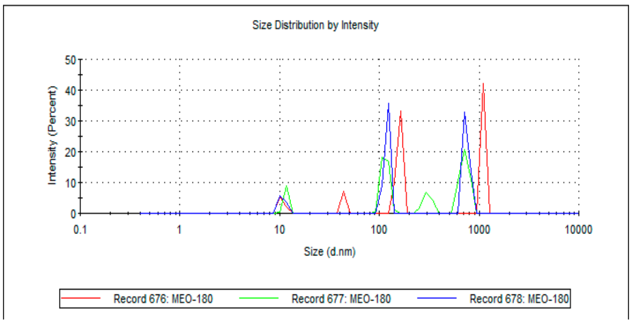

j´

			Mean (mV)	Area (%)	St Dev (mV)
Zeta Potential (mV):	-7,87	Peak 1:	-7,87	100,0	3,96
Zeta Deviation (mV):	3,96	Peak 2:	0,00	0,0	0,00
Conductivity (mS/cm):	0,155	Peak 3:	0,00	0,0	0,00

Result quality : Good

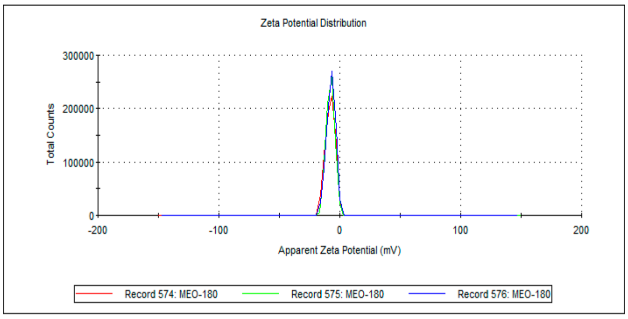

k´

			Size (d.nm):	% Intensity:	St Dev (d.nm):
Z-Average (d.nm):	413,6	Peak 1:	245,8	75,0	15,37
PdI:	0,763	Peak 2:	8,882	25,0	0,7388
Intercept:	1,01	Peak 3:	0,000	0,0	0,000

Result quality : Refer to quality report

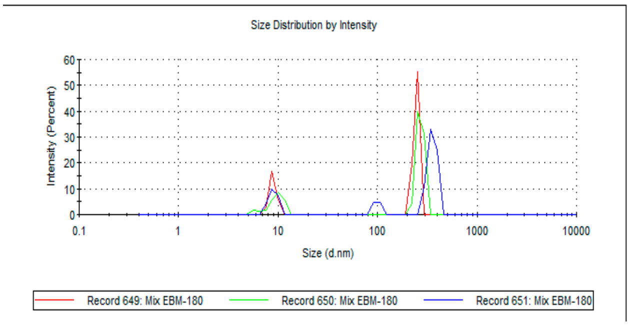

l´

			Mean (mV)	Area (%)	St Dev (mV)
Zeta Potential (mV):	-8,39	Peak 1:	-8,39	100,0	3,92
Zeta Deviation (mV):	3,92	Peak 2:	0,00	0,0	0,00
Conductivity (mS/cm):	0,0414	Peak 3:	0,00	0,0	0,00

Result quality : Good

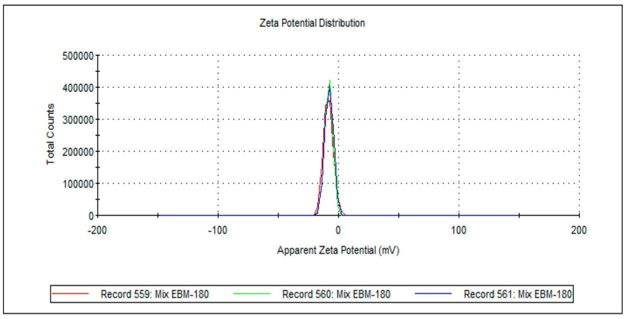

m´

			Size (d.nm):	% Intensity:	St Dev (d.nm):
Z-Average (d.nm):	213,7	Peak 1:	9,945	59,4	0,8982
PdI:	0,481	Peak 2:	162,2	40,6	6,310
Intercept:	0,998	Peak 3:	0,000	0,0	0,000

Result quality : Refer to quality report

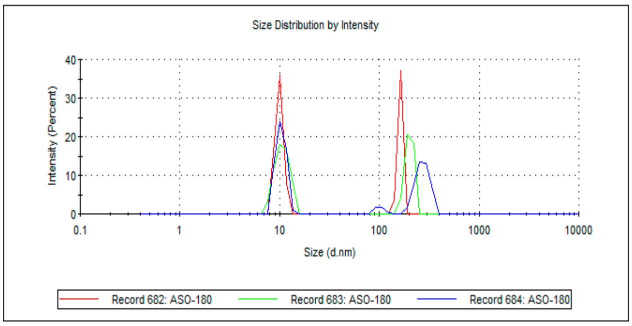

n´

			Mean (mV)	Area (%)	St Dev (mV)
Zeta Potential (mV):	-11,7	Peak 1:	-11,8	99,7	5,72
Zeta Deviation (mV):	5,84	Peak 2:	11,7	0,3	1,37
Conductivity (mS/cm):	0,0439	Peak 3:	0,00	0,0	0,00

Result quality : Good

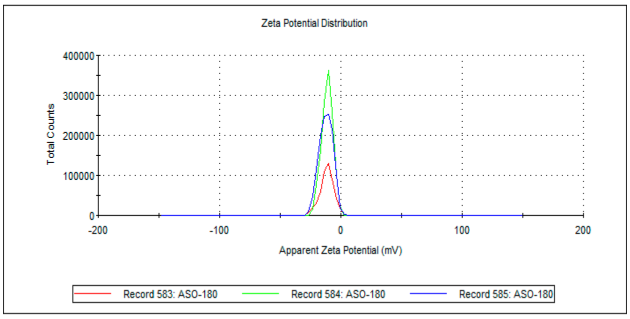

o´

			Size (d.nm):	% Intensity:	St Dev (d.nm):
Z-Average (d.nm):	982,3	Peak 1:	140,2	61,6	5,345
PdI:	1,000	Peak 2:	8,896	38,4	0,4596
Intercept:	1,18	Peak 3:	0,000	0,0	0,000

Result quality : Refer to quality report

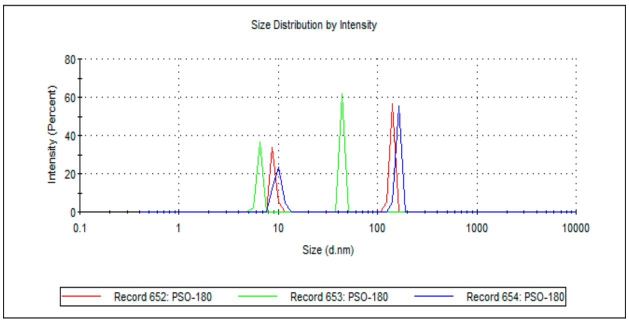

p´

			Mean (mV)	Area (%)	St Dev (mV)
Zeta Potential (mV):	-2,52	Peak 1:	-2,52	100,0	3,12
Zeta Deviation (mV):	3,12	Peak 2:	0,00	0,0	0,00
Conductivity (mS/cm):	0,129	Peak 3:	0,00	0,0	0,00

Result quality : Good

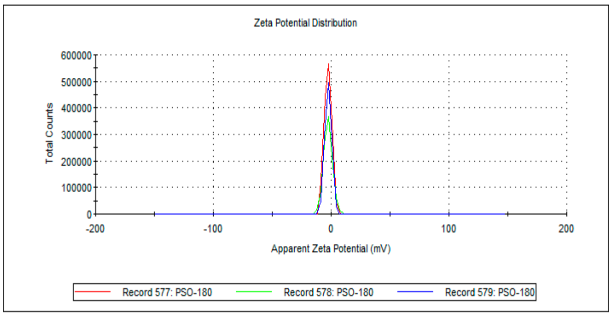

q´

			Size (d.nm):	% Intensity:	St Dev (d.nm):
Z-Average (d.nm):	104,3	Peak 1:	438,5	69,1	117,6
PdI:	1,000	Peak 2:	26,60	13,3	4,853
Intercept:	0,925	Peak 3:	14,13	10,3	2,036

Result quality : Refer to quality report

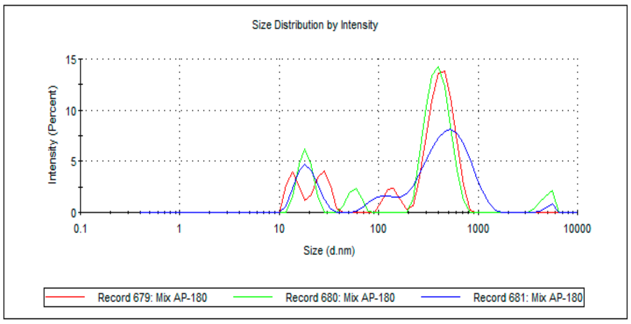

r´

			Mean (mV)	Area (%)	St Dev (mV)
Zeta Potential (mV):	-2,39	Peak 1:	-2,39	100,0	3,96
Zeta Deviation (mV):	3,96	Peak 2:	0,00	0,0	0,00
Conductivity (mS/cm):	0,0661	Peak 3:	0,00	0,0	0,00

Result quality : Good

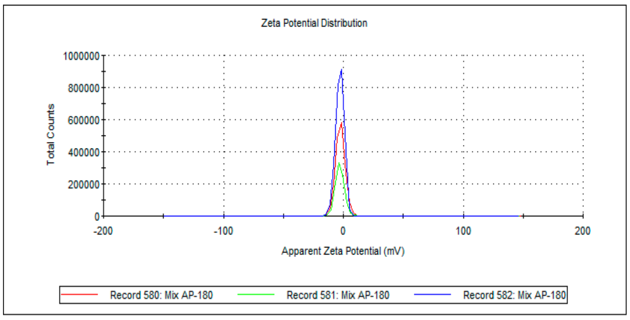

s´

			Size (d.nm):	% Intensity:	St Dev (d.nm):
Z-Average (d.nm):	277,4	Peak 1:	264,7	67,6	49,77
PdI:	0,621	Peak 2:	903,8	26,9	159,4
Intercept:	0,924	Peak 3:	13,26	5,5	1,389

Result quality : Refer to quality report

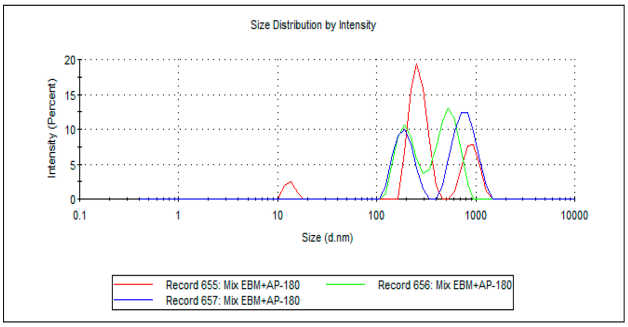

t´

			Mean (mV)	Area (%)	St Dev (mV)
Zeta Potential (mV):	-12,0	Peak 1:	-12,0	100,0	3,78
Zeta Deviation (mV):	3,78	Peak 2:	0,00	0,0	0,00
Conductivity (mS/cm):	0,164	Peak 3:	0,00	0,0	0,00

Result quality : Good

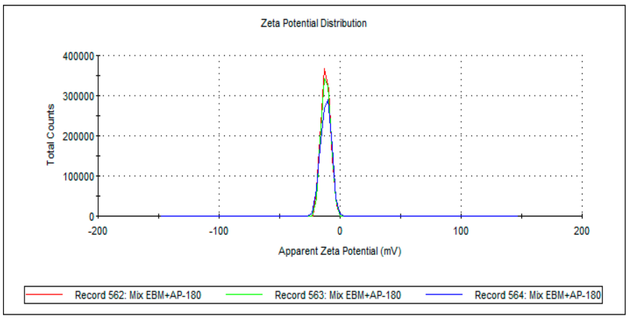

u´

Figure S1. Characterization of nanoemulsions performed with the dynamic light scattering method. The images show the hydrodynamic radius (droplet size), the polydispersity index (PDI) and the ζ-potential of the nanoemulsions stored during 0, 30 and 180 days of storage at 4 °C. The nanoemulsions were prepared from the oils of Eucalyptus (EEO); Basil (BEO); Mandarin (MEO); mix essential oils of Eucalyptus, Basil and Mandarin (Mix EBM); Avocado (ASO); Pumpkin (PSO); mix seed oils of Avocado and Pumpkin (Mix AP); mix of essentials and seed oils EBM+AP. The characteristics of the different nanoemulsions can be seen in the images with their letters in parentheses as follows: EEO in 0 (**a,b**), 90 (**p,q**) and 180 days (**f´,g´**); BEO in 0 (**c,d**), 90 (**r,s**) and 180 days (**h´,i´**); MEO in 0 (**e,f**), 90 (**t,u**) and 180 days (**j´,k´**); Mix EBM in 0 (**g,h**), 90 (**v,w**) and 180 days (**l´, m´**); ASO in 0 (**i,j**), 90 (**x,y**) and 180 days (**n´,o´**); PSO in 0 (**k,l**), 90 (**z,a´**) and 180 days (**p´,q´**); Mix AP in 0 (**m,n**), 90 (**b´,c´**) and 180 days (**r´,s´**); Mix EBM+AP in 0 (**ñ,o**), 90 (**d´,e´**) and 180 days (**t´,u´**).
