# Supplementary material for: Antibacterial Activity of Nanoemulsions Prepared with Essential and Seed Oils Against Isolated Bacteria from Rainbow Trout (Oncorhynchus mykiss)
Source: Foods. 2026 Jul 2;15(13):2340. doi: 10.3390/foods15132340 (PMC13361915; doi:10.3390/foods15132340)
Supplement: Supplementary file 1 [file foods-15-02340-s001.zip › Figure S2.pdf]

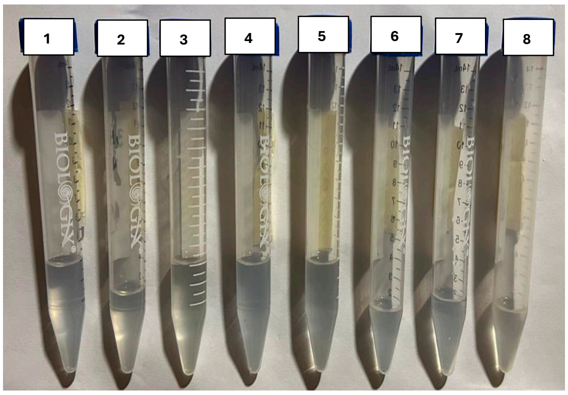

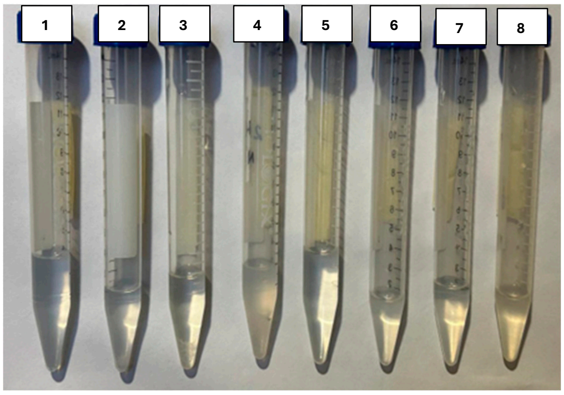

a

b

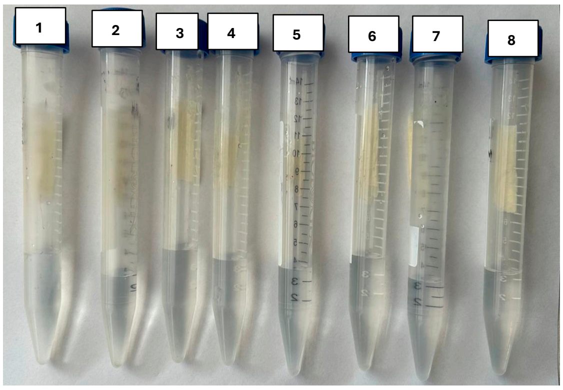

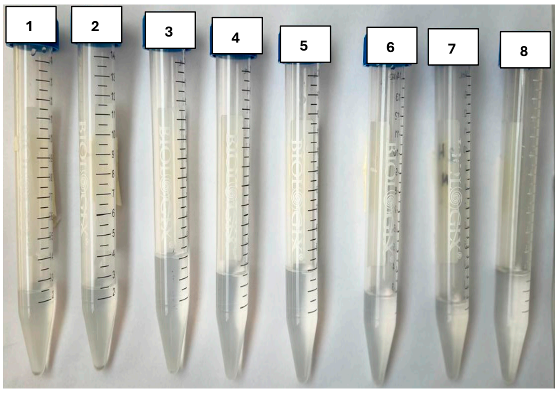

c

d

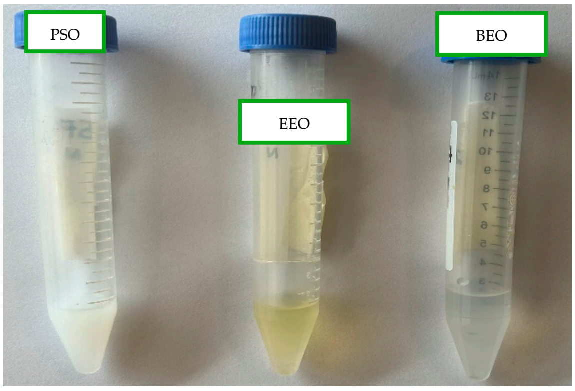

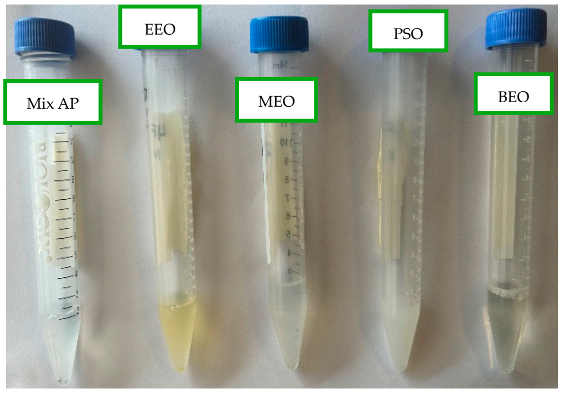

e

f

Figure S2. Thermodynamic stability of nanoemulsions stored at 4°C for 180 days. The photographs labeled **a, b, c**, and **d** indicate the thermodynamic stability of the eight nanoemulsions (**1** = essential oil mixture - Mix EBM. **2** = seed oil mixture - Mix AP. **3** = combination of the two above - Mix EBM+AP. **4** = eucalyptus – EEO. **5** = mandarin -MEO. **6** = basil-BEO. **7** = avocado-ASO. **8** = pumpkin-PSO) to centrifugation tests, heating and cooling cycles, and freeze-thaw stress during 0, 15, 30 and 90 days

of storage at 4 °C, respectively. The image labeled **e** shows the NEs that presented creaming and other alterations such as sedimentation and flocculation with the centrifugation test at 180 days, while the illustration labeled **f** shows the low stability of the NEs to the heating and cooling cycle and freeze-thaw stress during 180 days of storage.
